# Potent and specific MTH1 inhibitors targeting gastric cancer

**DOI:** 10.1038/s41419-019-1665-3

**Published:** 2019-06-04

**Authors:** Wenjuan Zhou, Liying Ma, Jing Yang, Hui Qiao, Lingyu Li, Qian Guo, Jinlian Ma, Lijuan Zhao, Junwei Wang, Guozhong Jiang, Xiangbin Wan, Mariusz Adam Goscinski, Lina Ding, Yichao Zheng, Wencai Li, Hongmin Liu, Zhenhe Suo, Wen Zhao

**Affiliations:** 1 State Key Laboratory of Esophageal Cancer Prevention and Treatment; Key Laboratory of Advanced Pharmaceutical Technology Ministry of Education of China; School of Pharmaceutical Sciences, Zhengzhou University, 100 Kexue Avenue, Zhengzhou, Henan 450001 China; 2 Department of Pathology, Oslo University Hospital, Faculty of Medicine, University of Oslo, Oslo, 0379 Norway; 3 Department of Pathology, The First Affiliated Hospital, Zhengzhou University, Zhengzhou, Henan 450001 China; 4 Department of General Surgery, Henan Provincial People’s Hospital, Zhengzhou, Henan 450001 China; 5 Department of Urology, The Norwegian Radium Hospital, Oslo University Hospital, Oslo, 0379 Norway

**Keywords:** Targeted therapies, Gastric cancer

## Abstract

Human mutT homolog 1(MTH1), the oxidized dNTP pool sanitizer enzyme, has been reported to be highly expressed in various malignant tumors. However, the oncogenic role of MTH1 in gastric cancer remains to be determined. In the current study, we found that MTH1 was overexpressed in human gastric cancer tissues and cells. Using an in vitro MTH1 inhibitor screening system, the compounds available in our laboratory were screened and the small molecules containing 5-cyano-6-phenylpyrimidine structure were firstly found to show potently and specifically inhibitory effect on MTH1, especially compound MI-743 with IC_50_ = 91.44 ± 1.45 nM. Both molecular docking and target engagement experiments proved that MI-743 can directly bind to MTH1. Moreover, MI-743 could not only inhibit cell proliferation in up to 16 cancer cell lines, especially gastric cancer cells HGC-27 and MGC-803, but also significantly induce MTH1-related 8-oxo-dG accumulation and DNA damage. Furthermore, the growth of xenograft tumours derived by injection of MGC-803 cells in nude mice was also significantly inhibited by MI-743 treatment. Importantly, MTH1 knockdown by siRNA in those two gastric cancer cells exhibited the similar findings. Our findings indicate that MTH1 is highly expressed in human gastric cancer tissues and cell lines. Small molecule MI-743 with 5-cyano-6-phenylpyrimidine structure may serve as a novel lead compound targeting the overexpressed MTH1 for gastric cancer treatment.

## Introduction

Reactive oxygen species (ROS), the by-products of cell metabolism, can suppress tumor growth by the oxidative damage of biological macromolecules, such as DNA, RNA, proteins and lipids^1–3^. The most abundant forms of ROS-related cellular damage in nucleotide pool are 8-oxo-dGTP and 2-OH-dATP^4–6^; Human mutT homolog 1 (MTH1, Nudix hydrolase 1, NUDT1), also known as human 8-oxoguaninenucleoside triphosphatase like mutT in E. coli, efficiently converts the mutagenic, oxidized purine dNTPs to their monophosphate forms and prevents their incorporation into nuclear and mitochondrial DNA^7–9^.

Recently, the levels and the catalytic activity of MTH1 have been found to be elevated in various cancer cell lines^10^ and many kinds of clinical specimens, including lung cancer^11^, brain tumor^12–14^, renal cancer^15^, breast cancer^16^, colorectal cancer^17^, non-small-cell lung cancer^18,19^, multiple myeloma^20^ and esophageal cancer^21^. Importantly, MTH1 expression is correlated with advanced cancer stage, tumor invasion and poor prognosis in some of those solid tumors^11–14,19–21^, but dispensable for normal cells^22^. These findings have led to the development of MTH1 inhibitors to treat those cancers. In 2014, two MTH1 inhibitors(TH588 and (S)-crizotinib) were firstly reported^10,23^ and recognized as novel small-molecule inhibitors to effectively target this protein and exhibit good anti-proliferation effects in various cancer cells(U2OS, Hela, MCF7, MDA-MB-231, SW480, SW620, DU145 and A549). Subsequently, in a number of articles, researchers further confirmed that either inhibiting MTH1 activity or downregulating its expression is associated with significant cancer cell death^14,16,24–33^, indicating the important role of MTH1 in cancer cell proliferation. In 2016, Kettle^34^ and Petrocchi et al,^35^ discovered some new potent and selective MTH1 inhibitors(compounds 15, 19, 24 and IACS-4759). Unfortunately, these compounds failed to exhibit good anti-cancer effects, making people doubt the vital effects of MTH1 in promoting cancer cell growth. A following article reported that the cytoxicity of TH588 may be related to its off-target effects to tubulin rather than MTH1^36^. To further validate the concept of specific MTH1 inhibitor for cancer treatment, Helleday et al.^37–40^ then described a new best-in-class MTH1 inhibitor (TH1579, Karonudib) (WO2015187088A1), which has been approved for phase I clinical testing in cancer patients with advanced solid malignancies (NCT03036228). They found that this compound could result in MTH1 downstream 8-oxo-dG accumulation, DNA damage and cell apoptosis, without targeting tubulin. They further proved that the failure of cytotoxicity of those MTH1 inhibitors reported above (compounds 15, 19, 24 and IACS-4759) may be due to their failure to introduce the toxic 8-oxodG lesion into DNA, indicating that whether there is 8-oxodG accumulation after targeting MTH1 may play a key role in mediating those compounds’ cytotoxicity. Based on these studies, MTH1 may be still a promising and attractive anti-cancer target.

Gastric cancer(GC) is the fifth most frequently diagnosed cancer and third leading cause of cancer-related deaths in developed countries^41^. Moreover, Gastric cancer is a highly heterogeneous disease, its etiology multifactorial, with complex host genetic and environmental factors contributing to its development^42–45^. The main etiological factors in gastric cancers are related to the overproduction of ROS and DNA damage, including diet, alcohol and tobacco habits, as well as Helicobacter pylori-induced inflammation^46^. Some human DNA repair genes, including *MTH1* have been reported to be highly expressed in digestive tract tumors^47,48^, indicating the possible involvement of MTH1 in the progress of these tumors. However, there are very few reports about the effect of MTH1 on gastric cancer. Therefore, it becomes necessary to investigate the role of MTH1 in this cancer’s development and whether targeting MTH1 could be a novel therapeutic approach for this cancer.

In this study, we found that the expression level of MTH1 was significantly increased in human gastric tissues and cells, as well as esophageal and liver cancer cells. Moreover, a novel potent and specific MTH1 inhibitor MI-743 with 5-cyano-6-phenylpyrimidine structure was firstly found and it could obviously induce the MTH1-related 8-oxo-dG accumulation, DNA damage and proliferation inhibition in two MTH1 highly expressed gastric cancer cell lines. Our findings indicate that MTH1 plays an important role in these two gastric cancer cell lines’ growth and compound MI-743 may serve as a lead compound targeting the overexpressed MTH1 for gastric cancer treatment.

## Results

### Preferential increase of MTH1 expression in esophageal, liver and gastric cancer cell lines, and gastric cancer tissues

**Fig. 1:**
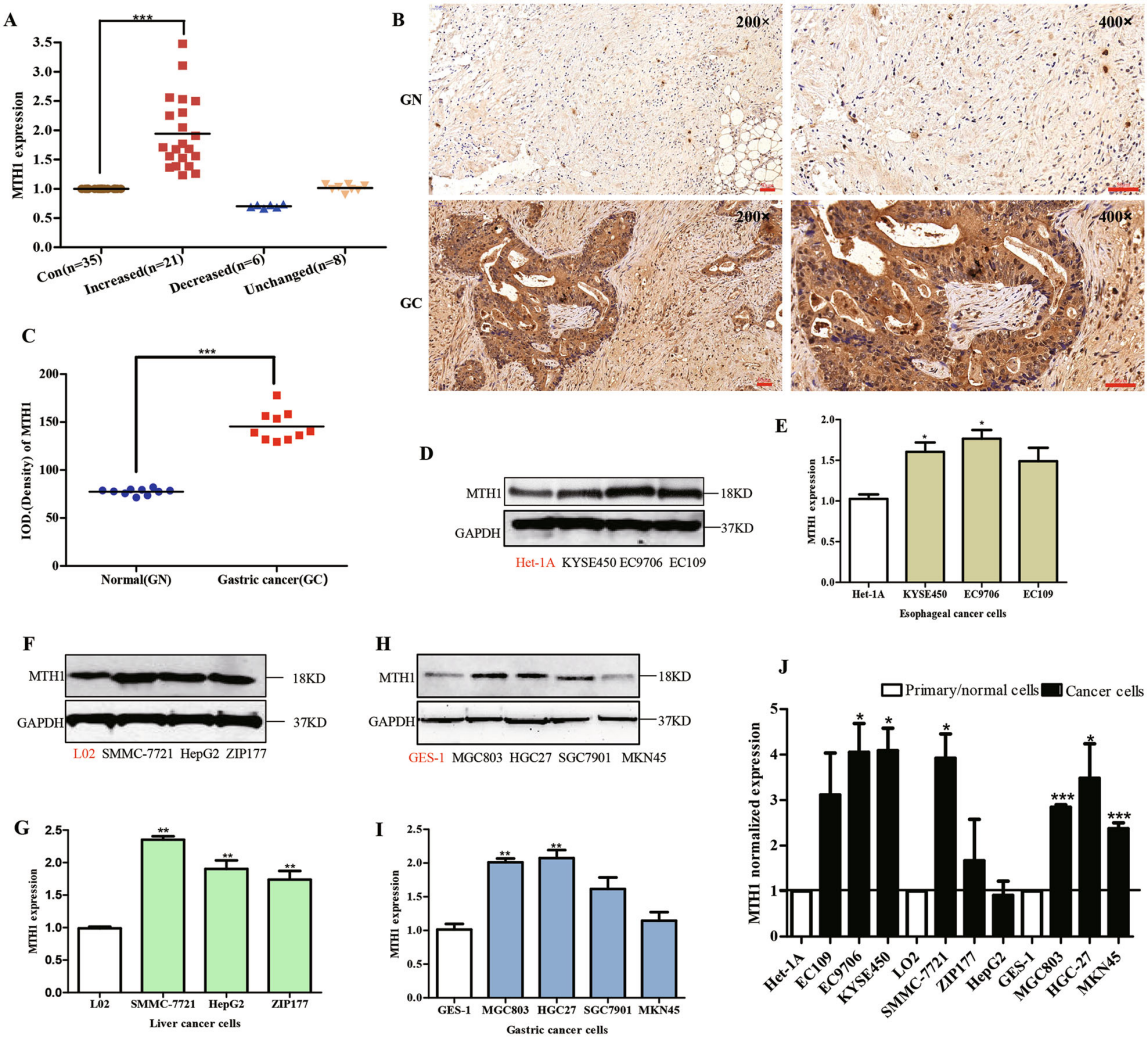
Characteristic expression of MTH1 in human gastric cancer tissues and ten digestive tract cancer cell lines. **a** The total RNA from fresh human gastric cancer tissues was isolated by RNApure Tissue&Cell Kit. The mRNA level of MTH1 in human gastric cancer (Increased, *n* = 21, Decreased, *n* = 6 and Unchanged, *n* = 8) and adjacent normal tissues (Con, *n* = 35) was determined by RT-PCR. The protein levels of MTH1 in human gastric (GC) and adjacent normal (GN) tissue sections were determined by immuohistochemistry (IHC) staining. The integral optical densities (IOD) were analyzed by Image-Pro Plus 6.0 software. **b** Representative IHC pictures of GC and GN. Scale bars, 50 μm. **c** IODs of GC and GN. *n* = 10 for each group. The cells from esophageal cancer cell lines: KYSE-450, EC109 and EC9706 (**d**, **e**), liver cancer cell lines: SMMC-7721, HepG2 and ZIP177 (**f**, **g**), gastric cancer cell lines: MGC-803, HGC-27, SGC-7901 and MKN45 (**h**, **i**), as well as the corresponding normal cell lines: Het-1A (**d**, **e**), L02 (**f**, **g**) and GES-1 (**h**, **i**) were cultured and lysed. The MTH1 protein levels were determined by Western Blot. GAPDH was used as a loading control. At least three independent experiments were performed for each group. **j** The cells indicated above were lysed and the total mRNA was extracted. The mRNA level of MTH1 was determined by RT-PCR. GAPDH was used as a control. At least three independent experiments were performed for each group. Data are presented as means ± SD. The symbol *, ** or *** stands for *P* < 0.05, *P* < 0.01 or *P* < 0.001 compared with the controls or normal cell groups

Firstly, the mRNA expression level of MTH1 in most (*n* = 21) of the human gastric cancer tissues (*n* = 35) was significantly increased to 2.04 folds, compared with those in their corresponding adjacent normal tissues (*n* = 35) (Fig. 1a). In addition, using immunohistochemistry, the expression level of MTH1 in the human tissue sections of gastric cancer (*n* = 10) and their corresponding adjacent normal sections (*n* = 10) was also determined. We found that the MTH1 was obviously increased to 1.83-folds in these tumor sections, compared with those in the adjacent normal sections (Fig. 1b, c). Our findings indicate that the expression level of MTH1 in human gastric cancer was highly overexpressed, which may play a role in this solid cancer’s development.

Furthermore, in the following cancer cell lines, including esophageal cancer cell lines (EC109, EC9706 and KYSE-450), liver cancer cell lines (SMMC-7721, HepG2 and ZIP177) and gastric cancer cell lines (MGC-803, HGC-27, SGC-7901 and MKN45), we found that MTH1 was highly expressed to 1.48-, 1.73-, 1.60-, 2.35-, 1.90-, 1.73-, 2.01-, 2.07-, 1.61-, 1.14-folds, compared with those in their corresponding normal cell lines(Het-1A, L02 and GES-1), respectively (Fig. 1d–i), the same as their mRNA expression levels shown in Fig. 1j. These findings suggest that MTH1 expression is also elevated in those tested cancer cell lines.

Furthermore, we searched the publicly available Oncomine database (https://www.oncomine.org) and TCGA (The Cancer Genome Atlas) database analytical website: UALCAN (http://ualcan.path.uab.edu/index.html)^49^ about the transcriptome data of NUDT1(MTH1) in stomach adenocarcinoma (STAD) (Supplementary Fig. 2A, B), esophageal carcinoma (ESCA) (Supplementary Fig. 2D) and liver hepatocellular carcinoma (LIHC) (Supplementary Fig. 2E). On the basis of these results, NUDT1 (MTH1) was also significantly overexpressed in these reported tumor tissues, compared to those with the corresponding non-tumor tissues. These findings are consistent with our results. Moreover, we found that in GC subtypes: Gastric Tubular Adenocarcinoma, Gastric Adenocarcinoma, Gastric Intestinal Type Adenocarcinoma, Diffuse Gastric Adenocarcinoma, Mucinous Gastric Adenocarcinoma, Signet Ring Cell Gastric Adenocarcinoma and Gastric Papillary Adenocarcinoma, MTH1 was also obviously overexpressed, compared with non-tumor gastric tissues, but there is no big difference among them (Supplementary Fig. 3A). In addition, from OncoLnc (www.oncolnc.org) website, it was found that the stomach adenocarcinoma (STAD) patients with high NUDT1 (MTH1) expression level exhibited a worse overall survival rate than those with low NUDT1 expression (Supplementary Fig. 2C), further indicating the possible negative correlation between MTH1 expression and the GC patients’ survival. However, from UALCAN, we found that there were no significant correlation between NUDT1 expression level and patients’ age and gender (Supplementary Fig. 3B, C).

### Screening of MTH1 inhibitors in vitro

**Fig. 2:**
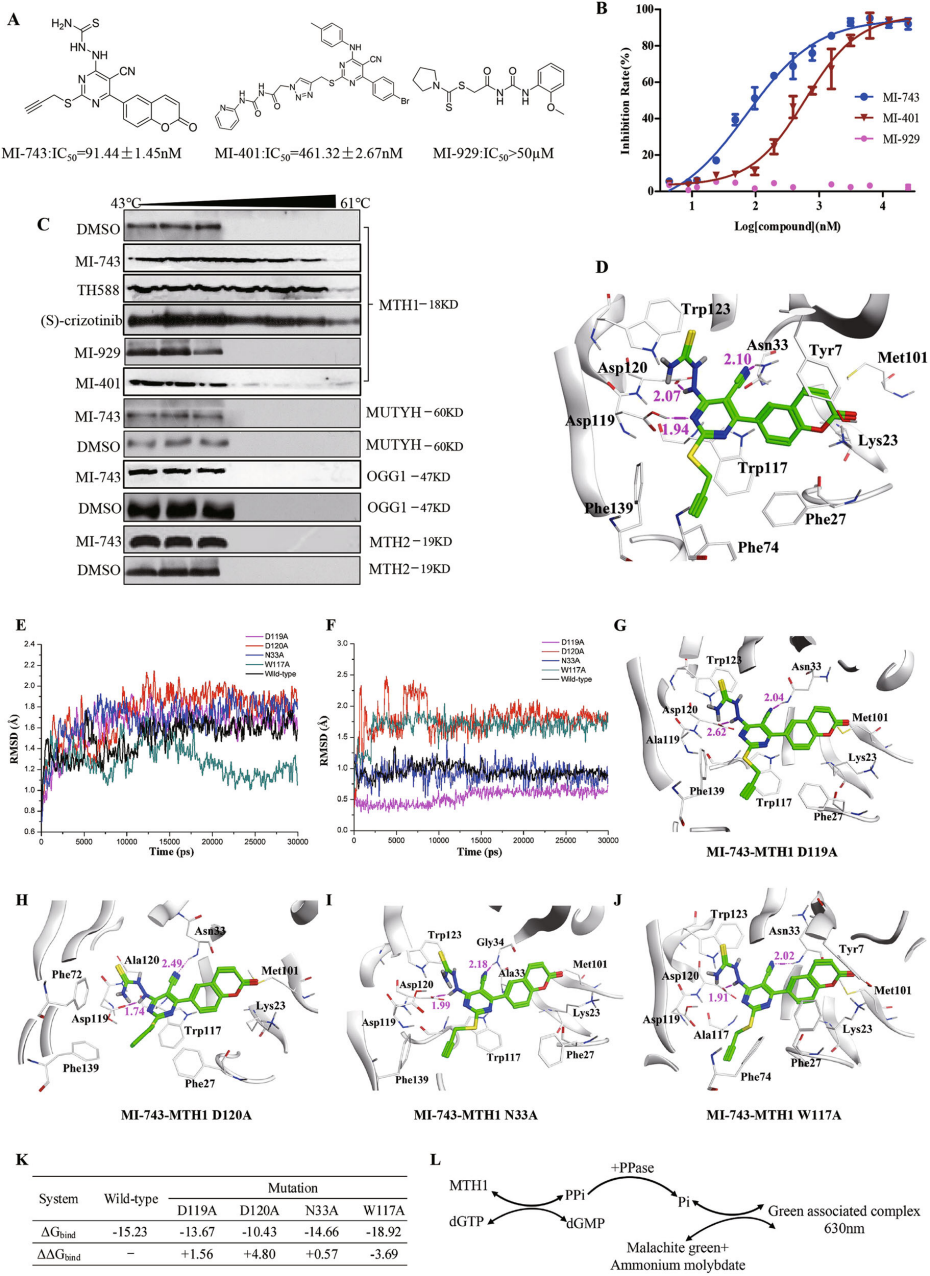
Screening of MTH1 inhibitors in vitro, MD simulations and binding free energy calculation of compound MI-743 and MTH1. **a** The structures, IC_50_ values and (**b**) percentage of MTH1 inhibition rate of the potential inhibitors MI-743, MI-401 and negative compound MI-929. **c** MGC-803 cells were treated with 50 µM compounds MI-743, MI-401, (S)-crizotinib or MI-929, respectively, collected and heated for 10 min from 43 to 61 °C. The cells were lysed and the protein levels of MTH1, OGG1, MUTYH or MTH2 were determined by western Blot. At least three independent experiments were performed for each group. Data are presented as means ± SD. **d** Predicted binding mode of compound MI-743 in MTH1 binding pocket (PDB code: 4N1U). Compound MI-743 is shown as green stick. The residues of MTH1 associated with MI-743 are shown in white. Hydrogen bonds are shown in magenta dash lines and the corresponding distances are given in Å. The RMSD value plots of the protein (MTH1) (**e**) and ligand (MI-743) (**f**) in the mutation and wild-type complex systems after 30 ns MD simulations. Binding models of compound MI-743 in MTH1 D119A (**g**), D120A (**h**), N33A (**i**) and W117A (**j**) mutations. **k** MM/PBSA binding free energy, estimated with the MTH1 wild-type and mutant systems obtained from the last 5 ns stable MD trajectory. **l** Flow diagram for the principle of detecting MTH1 activity. Since MTH1 can convert dGTP to dGMP and pyrophosphate, and the latter of which can be hydrolyzed to inorganic pyrophosphate by inorganic pyrophosphatase, the formed inorganic pyrophosphate can react with malachite green and ammonium molybdate to produce the green associated complex, the absorbance of which can be measured at 630 nm by PerkinElmer Envision microplate reader, indicating the activity of MTH1

Considering the elevated levels of MTH1 in our tested human tumor tissues and cancer cell lines, as well as the reported significance of this protein in cancer development^50^, small molecule inhibitors targeting MTH1 has been recognized as a novel target-based anti-cancer strategy^10,23^. Therefore, our following experiments were designed to setup compound screening system in vitro, specifically targeting MTH1. MTH1 recombinant protein and its mutant E56A were expressed and purified (Supplementary Fig. 4A). Then, the screening platform was optimized, using compound TH588 (Supplementary Fig. 4C) (IC_50_ = 10.23 ± 1.03 nM, compared to the reported IC_50_ = 5.0 ± 0.2 nM) as a positive control and the mutant protein E56A (Supplementary Fig. 4B) as a negative control^10^, respectively. Subsequently, the compounds (ca.1000 compounds) available in our laboratory were screened. We found that our compounds containing 5-cyano-6-phenylpyrimidine structure showed good inhibitory effect on MTH1. After further optimizing and screening, two potent compounds MI-743 (IC_50_ = 91.44 ± 1.45 nM) and MI-401 (IC_50_ = 461.32 ± 2.67 nM) were selected for the following experiments, with compound MI-929 (IC_50_ > 50 µM) as a negative control (Fig. 2a, b).

### Molecular docking compound MI-743 and MTH1

Next, molecular docking study was performed to investigate the specific binding mode of compound MI-743 in the active site of MTH1. In this study, two main docking conformations (named as type A, Fig. 2d and type B, Supplementary Fig. 5A) were formed for compound MI-743. Among them, 11 of 20 type As and 6 of 10 type Bs were recognized as top-score docking conformations, respectively. Thus, type A pose with the highest score (-10.495) was selected as the best docking conformation, which included some reported critical residues (Asp119, Asp120 and Asn33) in MTH1 active binding pocket^10,23,34,51^. As shown in Fig. 2d, the cyano group of compound MI-743 forms a hydrogen bond with Asn33 (2.10 Å) of MTH1. The amino group in aminothiourea connecting with pyrimidine ring forms a hydrogen bond with Asp120 (2.07 Å). Meanwhile, the nitrogen atom in pyrimidine ring forms a hydrogen bond with Asp119 (1.94 Å). The pyrimidine ring is found to have *π*–*π* stacking interaction with Trp117. The propargyl group occupies a hydrophobic cavity surrounded by Phe74, Phe139, Phe72, Phe27, Met81 and Gly141 residues. In addition, the coumarin moiety forms hydrophobic interactions with Phe27, Tyr7, Trp117, Met101 and Leu9 residues. All of these interactions indicate that compound MI-743 could be well docked into the active site of MTH1.

### MD simulations

To further determine the key residues of compound MI-743 binding in the active site of MTH1, the mutations of MTH1 (Asp119 to Ala119, Asp120 to Ala120, Asn33 to Ala33 and Trp117 to Ala117) were carried out before molecular docking study, respectively. After the mutations, MD simulations between compound MI-743 and the wild-type and mutant systems were applied to obtain stable conformations. As shown in Fig. 2e, f, the root mean square deviation (RMSD) results of protein (MTH1) from the wild-type and mutant systems and ligand (MI-743) tend to be stable after 30 ns, which suggested that the systems were equilibrated. Four stable conformations of the mutant systems were shown in Fig. 2g–j. In the modeled compound MI-743-MTH1 D119A, D120A, N33A and W117A complexes, the number of the hydrogen bonds are decreased, when compared with the wild-type model. These findings indicate that the residues of Asp119, Asp120, Asn33 and Trp117 of MTH1 may be crucial for binding with compound MI-743.

Furthermore, the binding free energies of wild-type and D119A, D120A, N33A, W117A mutations on structure of compound MI-743-MTH1 complexes were calculated by MM/PBSA module in AmberTools14 package. From the results of MM/PBSA estimate (Fig. 2k), the affinities of MI-743 binding with the D119A, D120A, and N33A mutated MTH1 were decreased to 89.75, 68.48 and 96.25%, this value was increased to 1.24 folds in the W117A mutated MTH1 system, which suggested that Asp119, Asp120 and Asn33 of MTH1 were crucial residues when binding to compound MI-743.

In addition, molecule docking of MI-401 and MTH1 was also performed. Similar to MI-743, MI-401 can form hydrogen bonds and *π*–*π* stacking interaction with MTH1 (Supplementary Fig. 5B). However, a long chain connecting with the pyrimidine ring is located out of the ligand-binding pocket (Supplementary Fig. 5C), which cannot adequately occupy the binding pocket. Therefore, MI-401 shows moderate activity when compared with MI-743. Moreover, the binding free energies (ΔG_bind_) predicted for MI-401-MTH1 complex are decreased (Supplementary Fig. 5D), compared with those values in MI-743-MTH1, further indicating the different binding activity between compounds MI-743 or MI-401 and MTH1.

### Compound MI-743 is specific for targeting MTH1 in cells

In order to further testify the specificity of compounds MI-743 and MI-401 on MTH1 rather than other 8-oxo-dGTP scavengers: human mutT homolog 2 (MTH2), human 8-oxoguanine glycosylase 1 (OGG1) and human mutY homolog (MUTYH) at cellular level, the cellular thermal shift assay (CETSA) was performed. As show in Fig. 2c, after compound MI-743 treatment, MTH1 was particularly stable until the incubation temperature was increased to 61 °C, similar to that of the positive compound TH588 or (S)-crizotinib^23^ treatment. However, after compound MI-401 or MI-929 treatment, the temperature of MTH1 degradation was significantly decreased to 49 °C, similar to the negative control (Fig. 2c), indicating a weak binding force between those two compounds and MTH1. However, other 8-oxo-dGTP scavengers including: MTH2, OGG1 and MUTYH became unstable at only 49 °C after MI-743 treatment, respectively. These findings indicated that compound MI-743 had specifically potent binding ability to MTH1 at cellular level without significant effects to the other similar proteins. Therefore, compound MI-743 was chosen to do the following cellular and in vivo experiments.

### Compound MI-743 causes cytotoxicity and induces 8-oxo-dG accumulation, DNA damage and apoptosis in MGC-803 and HGC-27 cells

**Fig. 3:**
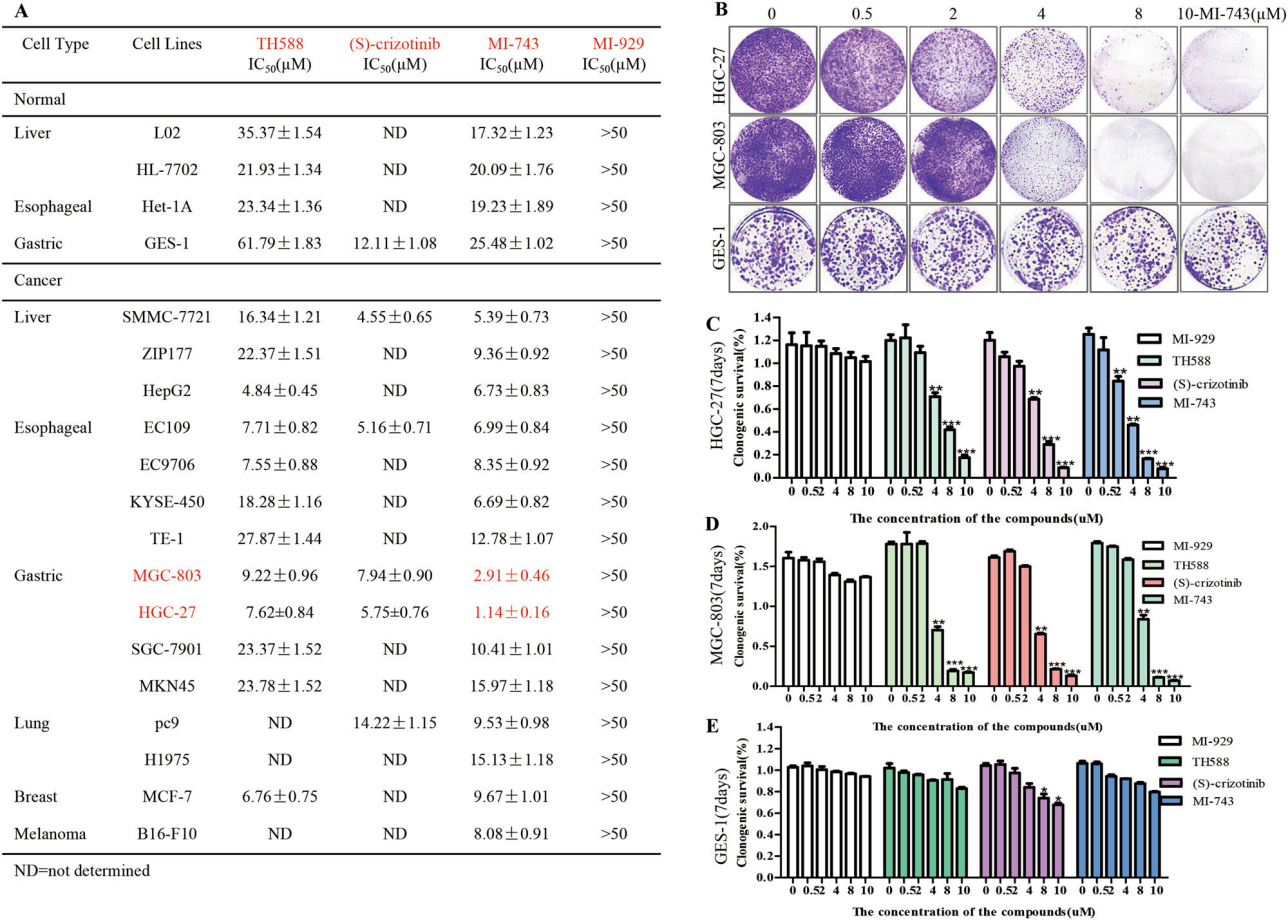
The cytotoxicities of compounds MI-743 and MI-929 on 16 cancer cell lines. **a** Four normal cell lines and above reported cancer cell lines were treated with different concentrations of MI-743 and MI-929 for 72 h. Cell viability was determined by MTT assay, using DMSO-treated controls. IC_50_ values are indicated as the means ± SD of three independent experiments. TH588 and (S)-crizotinib were used as positive control. **b** The colony formations of MGC-803, HGC-27 and GES-1 cells were determined after being treated with 0.5, 2, 4, 8 and 10 µM of compound MI-743. At least three independent experiments were performed for each group. **c**, **d** Clonogenic survival rates of gastric cancer cell lines after MI-743 and MI-929 treatments were determined by measuring the absorbance values at 595 nm in relation to DMSO-treated controls. TH588 and (S)-crizotinib were used as positive control. Data are presented as means ± SD. Three individual experiments were performed for each group. *P < 0.05, **P < 0.01, ***P < 0.001 as compared with the controls

Given that compound MI-743 can specifically target MTH1 at cellular level and in vitro, the cytotoxicity of MI-743 on cancer cells was measured, accompanied by compounds MI-929, TH588 and (S)-crizotinib. As shown in Fig. 3a, after treatment with these four compounds for 72 h, contrary to MI-929 but similar to TH588 and (S)-crizotinib, the cell viabilities of 16 cancer cell lines treated with MI-743 were sharply decreased, particularly human gastric cancer cell lines MGC-803 (IC_50_ = 2.91 ± 0.46 µM) and HGC-27 (IC_50_ = 1.14 ± 0.16 µM). However, it had little inhibitory effect on the 4 normal cell lines. Furthermore, colony formation assays were also performed, MGC-803 and HGC-27 cells, which were treated with MI-743, TH588 or (S)-crizotinib, formed smaller and fewer colonies, compared with those cells treated with DMSO or MI-929 (Fig. 3b–d). In addition, MI-743 had no obviously inhibitory effect on gastric normal cells (GES-1, Fig. 3b, e). Collectively, compound MI-743 exhibits significant cytotoxicity and anti-proliferation in up cancer cells, which may be related to MTH1 activity inhibition.

**Fig. 4:**
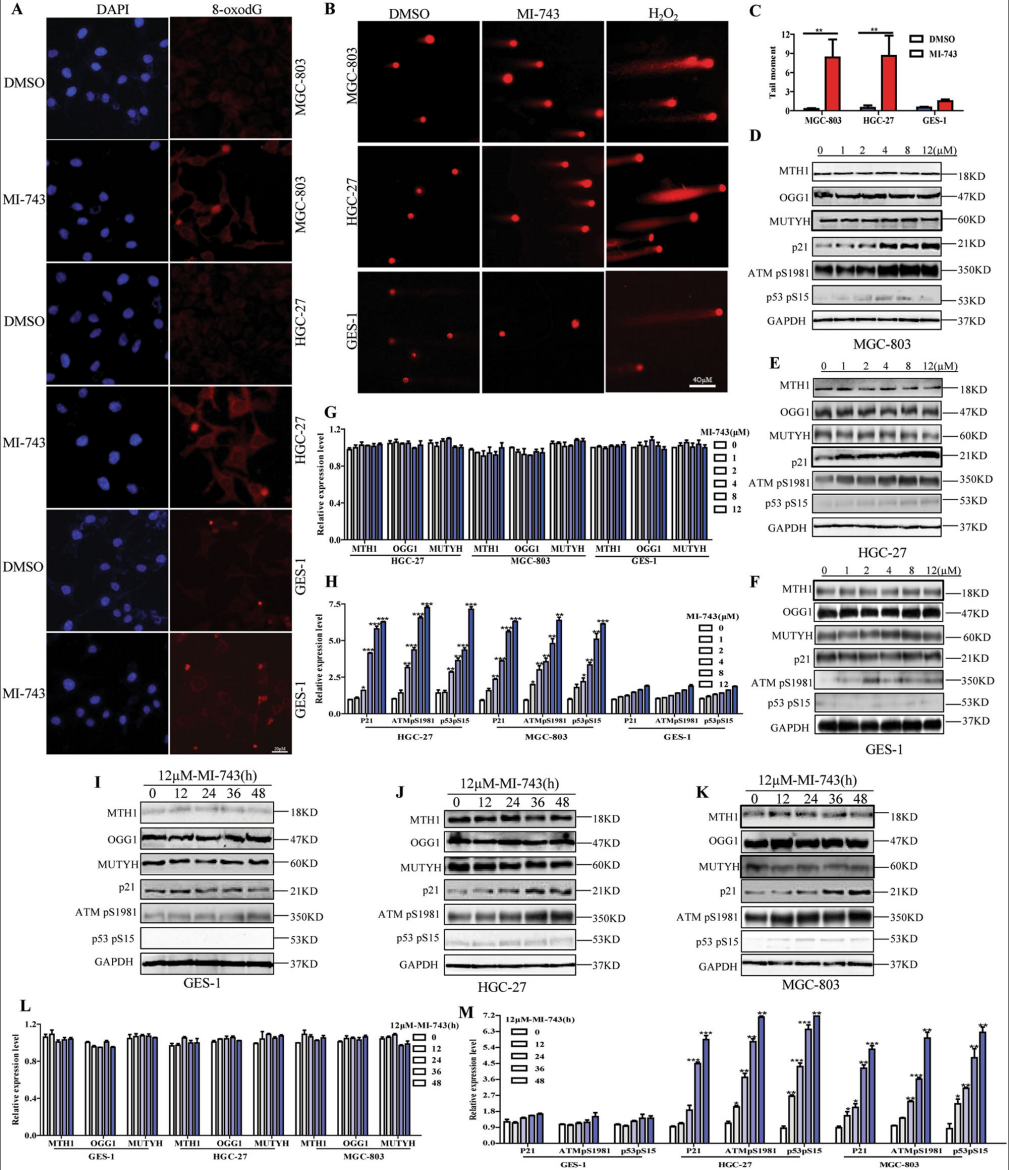
Compound MI-743 causes intracellular 8-oxo-dG accumulation and DNA damage. **a** MGC-803, HGC-27 and GES-1 cells were treated with DMSO or 5 µM MI-743 for 48 h. Intracellular 8-oxo-dG was stained with Cy3-conjugated avidin. DNA was counterstained with 4,6-diamidino-2-phenylindole (DAPI). Images were acquired at ×100 magnification by a Nikon Eclipse TE 2000-S fluorescence microscope. At least three independent experiments were performed for each group. **b** MGC-803, HGC-27 and GES-1 cells were treated with DMSO or 10 µM MI-743 for 48 h and run in alkaline comet assay. Pictures were originally captured at ×40 magnification. H_2_O_2_ was used as positive control. **c** Tail moment was calculated by CometScore software. Three individual experiments were performed for each group. **d**–**f** Western blot analysis of the protein levels of MTH1, MUTYH, OGG1, p21, ATMpS1981 and p53pS15 in MGC-803, HGC-27 and GES-1 cells, treated with increasing concentrations of MI-743 (0, 1, 2, 4, 8 and 12 µM). **g**, **h** Densitometry shows relative protein expression normalized for GAPDH. Data are representative of three independent experiments. **i**–**k** Western Blot analysis of the protein levels of MTH1, MUTYH, OGG1, p21, ATMpS1981 and p53pS15 of protein lysates, isolated from MGC-803, HGC-27 and GES-1 cells, which were treated with 12 µM MI-743 for 0, 12, 24, 36 and 48 h. **l**, **m** Densitometry shows relative protein expression normalized for GAPDH. Data are presented as means ± SD. Three individual experiments were performed for each group. *P < 0.05, **P < 0.01, ***P < 0.001 as compared with the controls

**Fig. 5:**
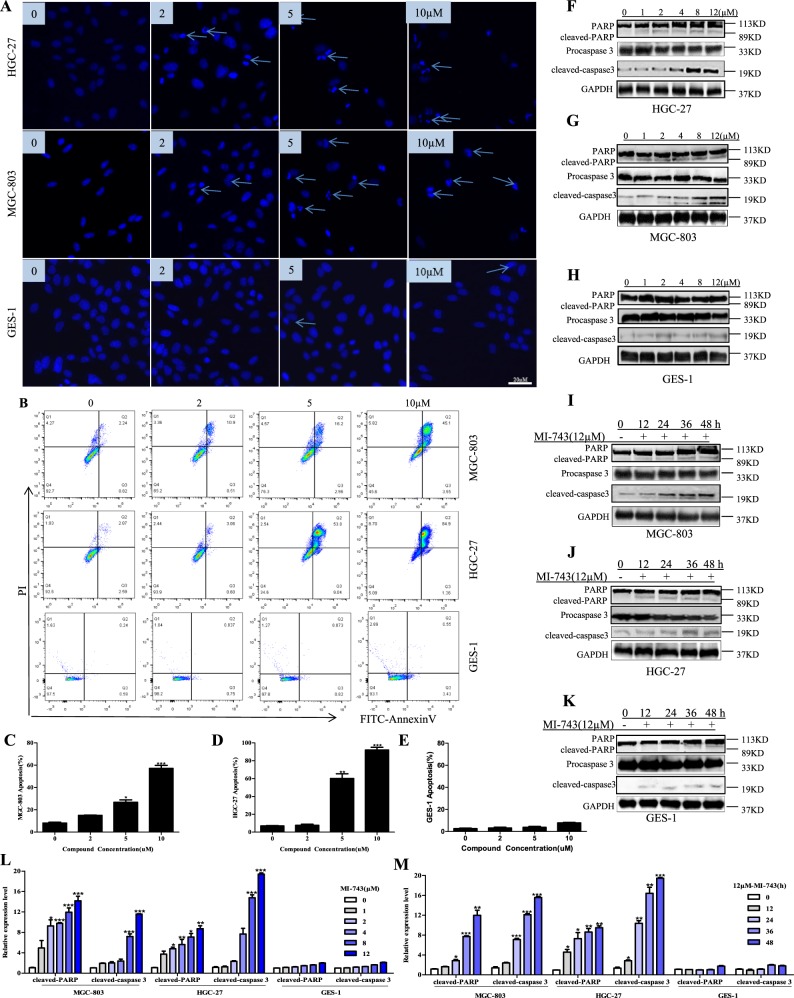
Compound MI-743 induces MGC-803 and HGC-27 cell apoptosis. MGC-803, HGC-27 and GES-1 cells were cultured with DMSO, 2, 5, 10 μM of MI-743 for 48 h. **a** Apoptosis-related nuclear condensation were stained with Hoechst 33258 and pictured by fluorescence microscopes. Pictures were originally captured at ×100 magnification. Representative photographs of three independent experiments were shown. **b**–**e** Apoptotic cells were detected using the Annexin V-FITC/ PI double staining and analyzed by flow cytometry in MGC-803, HGC-27 and GES-1 cells. The data were analyzed by FlowJo-V10 and Graph Pad Prism 5 software. Three individual experiments were performed for each group. **f**–**h** Western blot analysis of the protein levels of pro and cleaved-caspase 3, PARP and cleaved-PARP in MGC-803, HGC-27 and GES-1 cells, treated with increased concentrations of MI-743 (0, 1, 2, 4, 8 and 12 µM). **l** Densitometry shows relative protein expression normalized for GAPDH. Data are representative of three independent experiments. **i**–**k** Western Blot analysis of the protein levels of pro and cleaved-caspase 3, PARP and cleaved-PARP of protein lysates, isolated from MGC-803, HGC-27 and GES-1 cells, which were treated with 12 µM MI-743 for 0, 12, 24, 48 and 72 h. **m** Densitometry shows relative protein expression normalized for GAPDH. The data are presented as means ± SD. Three individual experiments were performed for each group. *P < 0.05, **P < 0.01, ***P < 0.001 as compared with the controls

Previously, it has been reported that inhibiting MTH1 can increase the content of genomic 8-oxo-dG, aggravate the incorporation of oxidized nucleotides into DNA, further induce DNA damage and apoptosis^10,37^. To further confirm the cellular effects of compound MI-743 are indeed related to MTH1 inhibition, we performed the following experiments. We found that the levels of 8-oxo-dG in MGC-803 and HGC-27 cells were sharply increased after being dealt with MI-743 for 48 h, compared with those in DMSO treatment (Fig. 4a). The values of comet tail moment were also significantly elevated, compared to those in DMSO control. 100 µM H_2_O_2_ treatment was used as a positive control to induce cellular oxidative stress (Fig. 4b, c). These findings indicate that the obvious DNA damage responses are induced by compound MI-743. Moreover, DNA damage response (DDR) signaling markers, including ATM-dependent phosphorylation of p53pS15 and ATMpS1981, as well as p53 target gene p21^10,37^, were tested. We found that after MI-743 treatment, the expression levels of p53pS15, ATMpS1981 and p21 were significantly increased dose and time-dependently without obvious effect on MTH1, MUTYH and OGG1 expression, compared to those in DMSO treatment (Fig. 4d–m). In addition, MGC-803 and HGC-27 cells, which were treated with MI-743, exhibited significant apoptosis-related morphologies, such as cell shrinkage, nuclear fragmentation and condensation, in a dose-dependent manner (Fig. 5a). Furthermore, the percentages of apoptotic cells and the cleaved-caspases 3 and PARP were markedly increased, without significant effects on GES-1 cells (Fig. 5b–m). These results suggest that compound MI-743 could markedly induce cellular DDR and apoptosis, which may be related to its specific inhibition to MTH1 activity.

### The antiproliferative activity of MTH1 inhibitor MI-743 on gastric cancer xenografts

**Fig. 6:**
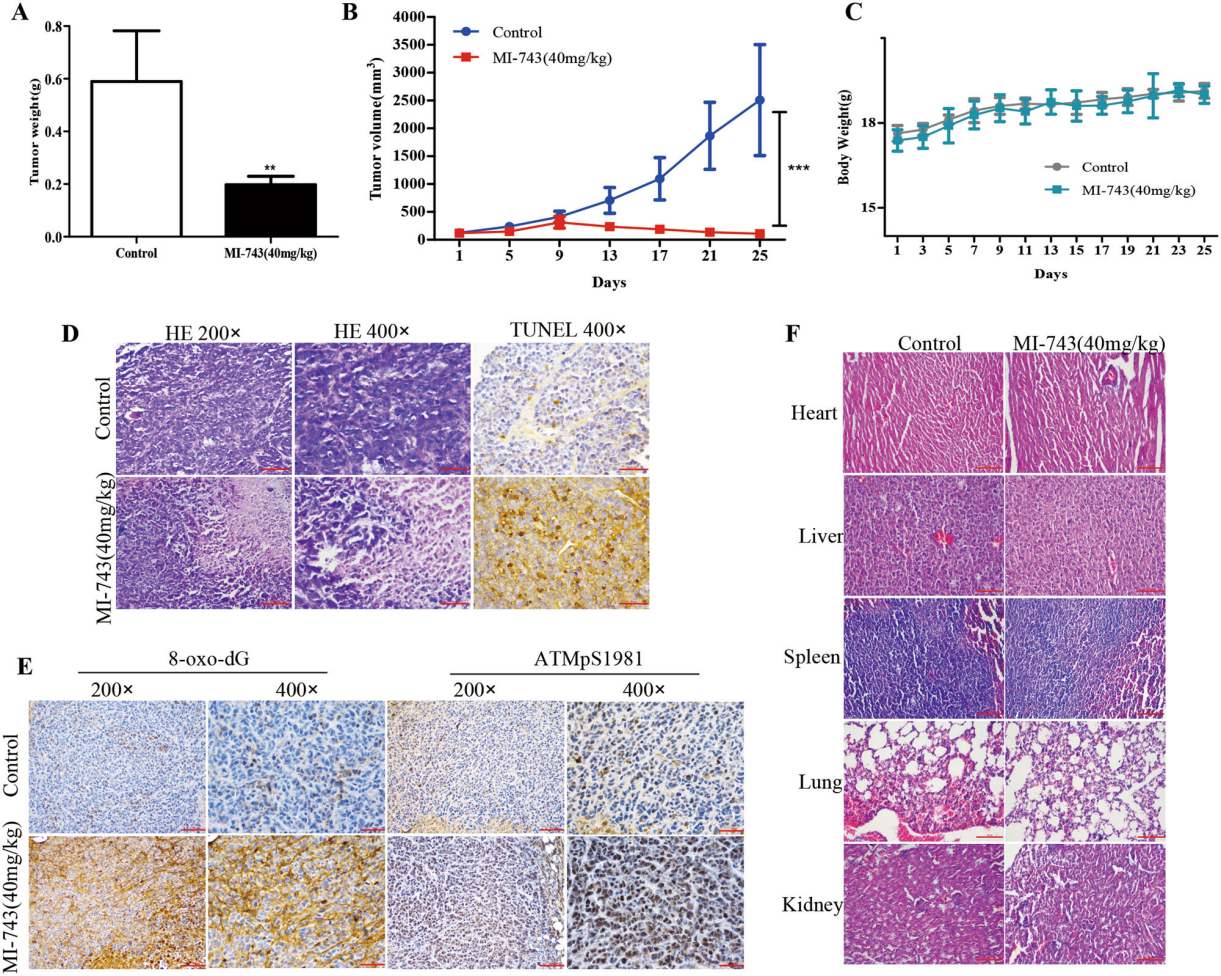
The anti-proliferative activity of MI-743 on gastric cancer xenografts. Gastric cancer cells MGC-803 were transplanted subcutaneously to the right side of BALB-C nude mice. When most of the tumor volumes reached 100 mm^3^, the mice were treated with oil or compound MI-743(40 mg/kg, once a day) for 25 days. **a** The average tumor weight. **b** The tumor volumes were measured every 4 days. (**c**) The mice body weights were measured every 2 days. **d**, **e** The tumor tissues were collected and fixed in 4% buffered paraformaldehyde and paraffin-embedded for immunohistochemistry to detect the protein level of 8-oxo-dG and ATMpS1981 (**e**), H&E staining to observe the cell morphology and TUNEL staining to examine apoptosis status (**d**). Pictures for immunohistochemistry and H&E staining were originally captured at ×200 and ×400 magnification. Pictures for TUNEL staining were originally captured at ×400 magnification. **f** The mice major organs were collected and fixed in 4% buffered paraformaldehyde and paraffin-embedded for H&E staining. Pictures were originally captured at 200 × magnification using microscope. *P < 0.05, **P < 0.01 or ***P < 0.001 compared with the controls

On the basis of the cellular effects of compound MI-743, its in vivo role on gastric cancer was further determined, using the MGC-803 cells-derived xenograft model in BALB/c nude mice. We found that the average tumor volumes in MI-743 treatment group (40 mg/kg for 25 days) were significantly decreased to 33.54% in comparison with those in the oil treatment group (Fig. 6a, b). However, the mouse body weight did not present obvious change among the two groups (Fig. 6c), indicating the less side effects of MI-743 on nude mice.

In addition, after MI-743 treatment, the expression levels of ATMpS1981 and 8-oxo-dG, were sharply increased (Fig. 6e). Moreover, massive area of cell destruction, cell death and significantly increased apoptotic cells were observed in the MI-743 treatment group, compared with those in the oil treatment group (Fig. 6d). In contrast, H&E staining of the liver, kidney, lung, heart and spleen showed no signs of toxicity by MI-743 treatment, compared with those in the oil treatment group (Fig. 6f). These data indicate that compound MI-743 was efficacious in inhibiting the MGC-803 cells growth in vivo without obvious global toxicity.

### The cellular effects of MI-743 is dependent on MTH1 in cancer cells

**Fig. 7:**
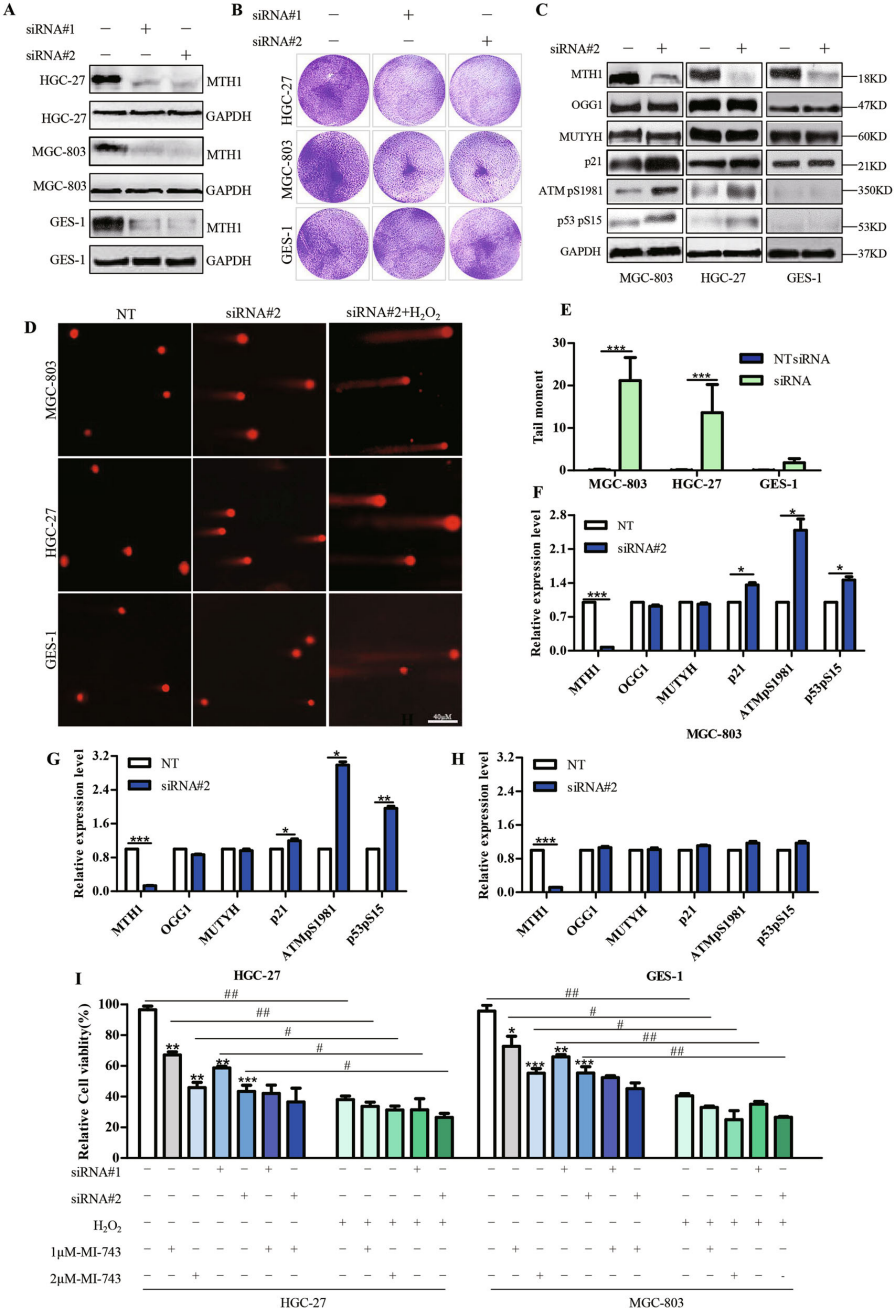
MTH1 suppression reduces the two gastric cancer cell survival. MGC-803, HGC-27 and GES-1 cells were treated by MTH1 specific siRNA#1 and #2 for 72 h. Non-targeting siRNA(NT) treatment was used as control. **a** Levels of MTH1 protein then were determined by western blot. **b** The colony formations were recorded after the cells were treated with MTH1 siRNA#1 and #2 for 7 days. **c** Western Blot analysis of the protein levels of MTH1, MUTYH, OGG1, p21, ATMpS1981 and p53pS15, after the cells were treated with siRNA#2. **f**–**h** Densitometry shows relative protein expression normalized by GAPDH. **d** The Alkaline comets assay was performed after the cells were transfected with MTH1 siRNA#2 for 72 h. Pictures were originally captured at ×40 magnification by fluorescence microscope. Non-targeting siRNA(NT) or H_2_O_2_ treatment was used as a negative or a positive control, respectively. **e** Tail moment was calculated by CometScore software. Three individual experiments were performed for each group. **i** Relative cell viability was determined by MTT assay, after the cells were treated with MTH1 siRNA#1 and #2 with or without MI-743 combined with 100uM H_2_O_2_. Data are presented as means ± SD. Three individual experiments were performed for each group. The symbols * and #, ** and ## or ***and ### stand for *P* < 0.05, *P* < 0.01 or *P* < 0.001 compared with the controls

Considering the elevated levels of MTH1 in various of cancer cell lines, including esophageal, liver and gastric cancer cells, and the possible relationship between MTH1 activity and the cytotoxic effects of compound MI-743, we hypothesized that MTH1 may be a key mediator in the consequence of MI-743 in these cancer cells. To confirm this, two different reported short interfering RNAs (siRNAs) targeting MTH1 were used to down-regulate MTH1 expression^10^. As show in Fig. 7a, b, i and Supplementary Fig. 6A, B, MTH1 depletion resulted in significantly decreased cell viability in most of the cancer cells, and DNA damage response (Fig. 7c–i) in MGC-803 and HGC-27 cells, without markedly proliferation inhibitory effects in GES-1 cells. In addition, down-regulation of MTH1 in MGC-803 and HGC-27 cells also enhanced the expression levels of the p53pS15, ATMpS1981, as well as p21, but didn’t change the expression of MUTYH and OGG1(Fig. 7c, f, g, h), similar to the previous findings^10,23,37^. And it is noteworthy that MI-743 treatment did not further obviously decrease the MGC-803 and HGC-27 cells viability, when the cells were pretreated with MTH1 siRNA#1 or siRNA#2 (Fig. 7i). Moreover, after depleting MTH1 by siRNA or treated by compound MI-743, both MGC-803 and HGC-27 cells became more sensitive to H_2_O_2_ treatment (Fig. 7i), indicating that the expression of MTH1 or its activity plays a key role in these two gastric cancer cells’ survival, and the cellular effects of MI-743 are indeed dependent on MTH1 in these two cancer cell lines.

## Discussion

**Fig. 8:**
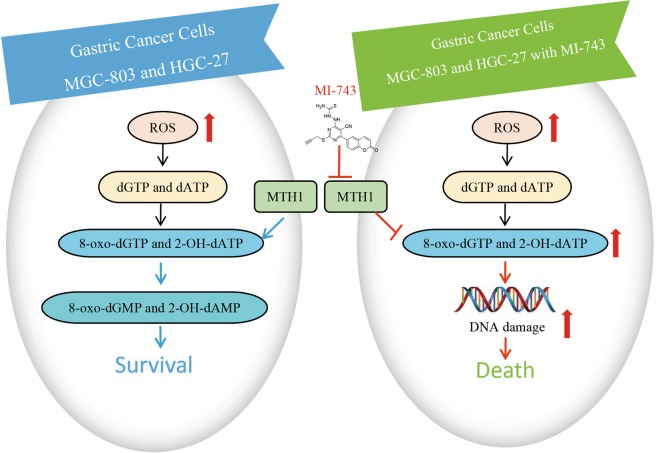
Proposed mechanisms for MI-743 induced cell death in gastric cancer cells MGC-803 and HGC-27 through targeting MTH1. In gastric cancer cells MGC-803 and HGC-27(left), increased ROS convert dGTP and dATP to 8-oxo-dGTP and 2-OH-dATP, respectively. The overexpressed MTH1 hydrolyzes the oxidized triphosphates into the corresponding 8-oxo-dGMP and 2-OH-dAMP, in order to prevent their incorporation into double strand DNA and further maintain cancer cell survival. However, under the treatment of compound MI-743(right), the enhanced activity of MTH1 in cancer cells is inhibited. The oxidized nucleotides (8-oxo-dGTP and 2-OH-dATP) will be incorporated into DNA, which further causes double strand DNA damage and finally induces cell death

In the current study, we demonstrate that MTH1 is highly expressed in human gastric cancer tissues and cells. Moreover, for the first time, we reported that a potent and specific MTH1 inhibitor MI-743 with 5-cyano-6-phenylpyrimidine structure could not only inhibit MTH1 activity in vitro and at cellular level, but also induce the intracellular MTH1-related 8-oxo-dG accumulation, leading to cell apoptosis. Our findings strongly suggest that targeting inhibition of the highly activated MTH1 and the resulting downstream 8-oxo-dG gathering play a crucial role in mediating MI-743-induced gastric cancer cell apoptosis. MI-743 and its scaffold may serve as a lead compound for the MTH1-related gastric cancer treatment (Fig. 8).

Gastric cancer, a common gastrointestinal malignancy worldwide, has high recurrence, poor prognosis and limited treatment options^45^. Except for the dysfunctional redox regulation due to chronically-hyperactivated mitogenic and pro-survival signaling, as well as metabolic alterations like all other cancer cells^52–54^, a variety of factors, including sodium chloride, Helicobacter pylori infection and smoking^55,56^, further induce the production of ROS in gastric cancer^57^. Such increased ROS production are thought to lead to 8-oxo-dG accumulation, DNA damage, carcinogenesis and the transcription or post-translational modification of base excision and other DNA repair enzymes in the gastric cancer^58,59^. Therefore, gastric cancer may have a greater reliance on these DNA repair enzymes’ function and targeting these enzymes, including MTH1, may be a valid strategy for gastric cancer treatment. So far, only a few papers reported that MTH1 exhibited increased mRNA expression levels in the gastric tumor specimens^50^. In the current study, we found the significantly enhanced MTH1 mRNA and protein levels in human gastric cancer tissues and two gastric cancer cell lines. Furthermore, knocking down MTH1 in these cell lines is associated with significantly decreased cell proliferation and DNA damage. These findings indicate that MTH1 indeed plays an important role in these gastric cancer cells’ growth, which are similar to those publications in cancer cell lines (U87MG, U251MG, Mel-CV, Mel-RMu, GBM line #7 and #18)^14,30,38^. However, recently, some articles described that down regulation of MTH1 in several cancer cell lines(HEMC, Hela, SW480, U2OS, HA549 and MCF7) could not inhibit these cells’ proliferation. Therefore, researchers began to doubt the importance of MTH1 for cancer treatment^34,36,60^. Indeed, Helleday et al.^10^ previously found that siRNA targeting MTH1 does not inhibit cell proliferation in some cell types, due to genetic or phenotypic resistance mechanisms and they speculated that MTH1 may be not necessary for all cancer cell growth^10,37^. Furthermore, the cytotoxicity of MTH1 inhibitors TH588 and TH1579 may be more efficient than siRNA-induced MTH1 protein loss, which may be related to the compensatory mechanism through MTH1’s gradual exhaustion^37^. Therefore, based on these studies, we speculated that the therapeutic effects of chemical inhibition and gene silence may not always work correlatively.

However, compared to the effects of TH588 and TH1579^10,37,38^, it is hard to explain that the reported potent MTH1 inhibitors^34,61^ could not incorporate the 8-oxodG into DNA and induce cancer cell death, even though they can engage in MTH1 at cellular level. However, Helleday et al.^8^ had speculated some reasons, but to answer these questions, it is necessary to plan more profound experiments to further determine the intracellular effects of MTH1. To prove the targeting effects of compound MI-743 on MTH1, we did a series of on-target evaluation experiments. The results suggest that MI-743 is highly selective in binding and inhibiting MTH1.

In conclusion, in the current study, we describe that MTH1 is highly expressed in gastric cancer tissues and two cell lines. Small molecule MI-743 with 5-cyano-6-phenylpyrimidine structure can specifically target MTH1 and inhibit the two gastric cancer cells growth both in vitro and in vivo. MI-743 may serve as a novel lead compound targeting the overexpressed MTH1 for gastric cancer treatment.

## Materials and Methods

### Chemicals, reagents and antibodies

Mouse monoclonal antibodies against anti-ATM (phospho S1981, ab81292) and anti-8-Oxoguanine(ab206461) antibody were purchased from Abcam Biotechnology (Cambridge, UK). Rabbit polyclonal antibodies against MTH1(NB100-109) was from Novus Biologicals. Rabbit polyclonal antibody against phospho-p53 (Ser15, 9284 T) was purchased from Cell Signaling Technologies. Rabbit polyclonal antibodies against MUTYH(19650-1-AP), OGG1(15125-1-AP), p21(10355-1-AP), PARP(13371-1-AP), and caspase 3(19677-1-AP) were purchased from Proteintech (Wuhan, China). Rabbit polyclonal anti-MTH2 antibody (bs-4401R) was purchased from Bioss biology Co., Ltd(Beijing, China).

RPMI-1640 and Fetal Bovine Serum were obtained from Hyclone Laboratories (Utah, USA). Ultrapure RNA Kit(CW0581), RNApure Tissue&Cell Kit(CW0584), HiFi-MMLV cDNA synthesis kit(CW0744) were purchased from CWbiotech(China). Fast Start Essential Universal DNA Green Master(06402712001, LightCycler® 8-Tube Strips(white) (06612601001) were purchased from Roche. E. coli DH5α and BL21(DE3) were obtained from Life technologies. Kanamycin, isopropyl-β-D-galactopyranoside(IPTG)(I8070), Bicinchoninic Acid (BCA) Protein Assay kit (PC0020), Hoechst 33258(B8030), 3-(4,5-dimethylthiazol-2-yl)-2,5-diphenyltetrazolium bromide (MTT) (M8180) were purchased from Solarbio(China). Ni-beads column were purchased from QIAGEN (USA). dGTP(D7355) and 4,6-diamidino-2-phenylindole(DAPI)(C1002) were purchased from Beyotime Biotechnology(China). Inorganic pyrophosphatase were purchased from Sigma. Malachite green (M110699), ammonium molybdate (A116378) and crystal violet solution (C110702) were purchased from Aladdin(China). Annexin-V-FITC/PI apoptosis kit(KGA106) were purchased from Keygen Biotech(China). Enhanced chemiluminescence(QL228436) and Lipofectamine® RNAiMAX Reagent(13778030) were purchased from Thermo Fisher Scientific. Cy3-conjugated avidin (0.5 µg/mL) (D111116-0100) was purchased from BBI Life Sciences.

### Cell lines

Human esophageal carcinoma cell lines EC109, EC9706, KYSE-450, gastric carcinoma cell lines MGC-803, HGC-27 and MKN45, and liver carcinoma cell lines HepG2 SMMC-7721 and ZIP177 were obtained from Cell Bank of the Chinese Academy of Sciences (Shanghai, China). Human immortalized normal esophageal epithelial cell Het-1A was a gift from the First Affiliated Hospital of Zhengzhou University, which was purchased from the American Type Culture Collection (Manassas, VA, USA). Human gastric carcinoma cell line SGC-7901 was purchased from Shanghai Bogoo Biotechnology Company. Human gastric epithelial mucosa cell line GES-1 and human breast cancer cell line MCF-7 were purchased from the State Key Laboratory of Molecular Oncology, Chinese Academy of Medical Sciences (Beijing, China). Cells were cultivated in corresponding medium supplemented with 10% FBS and 1% penicillin–streptomycin solution, at 37 °C with 5% CO_2_. All the cells were regularly tested the Mycoplasma infection.

### RNA isolation and quantitative RT-PCR analysis

Total RNA was isolated from cultured cell lines, including: normal cell lines L02, HL-7702(liver), Het-1A(esophageal), GES-1(gastric) and cancer cell lines SMMC-7721, ZIP177, HepG2(liver), EC109, EC9706, KYSE-450(esophageal), MGC-803, HGC-27, MKN45(gastric) and MCF-7(breast), using Ultrapure RNA Kit. Fresh human gastric cancer tissues were acquired from the Department of General Surgery of Henan Provincial People’s Hospital. The total RNA was isolated by RNApure Tissue&Cell Kit, which was designed specially to extract and purify RNA from cells and tissues respectively. After determining the purity of the isolated RNA by measuring UV absorption of each sample with comparable A260/A280 ratios, cDNA synthesis was performed using HiFi-MMLV cDNA synthesis kit. The RT-PCR reaction volume was 20 µl and the reaction systems included Fast Start Essential Universal DNA Green Master, cDNA, primers directed against MTH1, GAPDH, OGG1, MTH2, MUTYH and LightCycler® 8-Tube Strips(white). The specificity of the primer pairs (listed in Supplementary Fig. 1B) were initially tested by primer-BLAST and agarose gel electrophoresis and were synthesized by Liuhe Huada gene technology company (Beijing, China). Relative mRNA levels were calculated by BioRad software and normalized with the mRNA expression of GAPDH.

### Immunohistochemistry assay

Tissue sections (5 μm) of human esophageal squamous carcinoma and gastric carcinoma were acquired from the Department Pathology of the First Affiliated Hospital of Zhengzhou University. Prior to the tissue collection, the clinical protocol was approved by the ethics review board of the First Affiliated Hospital of Zhengzhou University. Informed written consents were obtained from the patients. Every specimen was handled anonymously, according to the ethical and legal standards. Deparaffinized sections with xylene and ethanol were treated with 3% hydrogen peroxidase (H_2_O_2_) for 5 min at room temperature to block endogenous peroxidase, then rinsed (3 × 5 min) in PBST (0.01 M PBS, 0.05% BSA, 0.0015% TritonX-100) and blocked with 5% BSA. Anti-MTH1 antibody was applied on the sections for 30 min at room temperature. After washing three times with PBS, the sections were incubated with secondary antibody for 30 min, followed by DAB staining for 10 min and then counterstained with hematoxylin and dehydrated. The sections were examined with an Olympus BX51 microscope (Olympus, Japan) and images were acquired with a camera (Olympus, Japan). The integral optical densities (IOD) were quantified by Image-Pro Plus 6.0 software.

### TCGA data retrieval and bioinformatics analysis

TCGA (The Cancer Genome Atlas) data were analyzed by TCGA database analytical websites: Oncomine (https://www.oncomine.org), OncoLnc (www.oncolnc.org) and UALCAN (http://ualcan.path.uab.edu/index.html)^49^.

### Expression and purification of MTH1 and MTH1 mutant E56A

The expression and purification of MTH1 was performed as described previously^10^. The plasmids pET28a-MTH1 and its mutant pET28a-E56A, the gifts from Dr. Thomas Helleday group in Karolinska Institute were transformed into E. coli BL21(DE3). A preculture was grown in 3 L medium containing 30 µg/ml kanamycin at 37 °C. After about 5 h, when the absorbance reached 0.7 at 600 nm, 0.25 mM isopropyl-β-d-galactopyranoside(IPTG) was added to induce the synthesis of His-tag-fusion proteins for 8 h at 20 °C. Subsequently, cells were collected and disrupted in 10 ml ice-cold washing buffer(10 mM Tris-HCl pH 7.4, 1 mM DTT, 500 mM NaCl) by sonication, the soluble and pellet fractions were separated by centrifugation at 12,000 g for 15 min. After filtration, the filtrate was applied to a Ni-beads column and washed with washing buffer containing different concentration of imidazole. The interested proteins were eluted with washing buffer(10 mM Tris-HCl pH 7.4, 1 mM DTT, 500 mM NaCl and 100/150 mM Imidazole) and the molecular weight of these proteins were confirmed by Commassie Blue staining (Supplementary Fig. 4A). The concentrations of the recombinant proteins were determined by a Bicinchoninic Acid (BCA) Protein Assay kit.

### Evaluation of the MTH1 recombinant protein activity in vitro and its inhibitors screening

On the basis of the ability of MTH1 in converting dGTP to dGMP and pyrophosphate, and the latter can then be hydrolyzed to inorganic pyrophosphate by inorganic pyrophosphatase, the MTH1 activity was designed to measure the product of inorganic pyrophosphate, after its reaction with malachite green and ammonium molybdate(Fig. 2l), the green associated complex was measured at 630 nm by PerkinElmer Envision microplate reader^10^.

Firstly, the assay buffer (100 mM Tris-acetate at pH 8.0, 40 mM NaCl, 10 mM Mg-acetate, 0.005% Tween 20 and 1 mM DTT) was added into a 96-well plate. Then, 5 nM recombinant human MTH1, 100 mM dGTP and 0.2U/ml inorganic pyrophosphates were added in sequence. After 60 min reaction, the mixture of 0.45 mg/mL malachite green and 4.2% ammonium molybdate in proportion of 3:1 were added. Then, the plates were incubated and shaken at room temperature for another 60 min. The absorbance was measured at 630 nm using the PerkinElmer Envision micro plate reader.

### Cellular Thermal Shift Assay

Cellular thermal shift assay (CETSA), also known as the target engagement assay was one-way to detect the intracellular interaction ability of compounds with its specific target^48^. Firstly, cells were seeded into T75 culture flasks and cultured overnight. Then, the cells were treated with cell media containing 1‰ DMSO as blank control and candidate compounds for 3–5 h. Next, the cells were collected by centrifugation and resuspended by PBS, then the cells were aliquoted into 7 PCR tubes and heated using PCR instrument for 10 min from 43 to 61 °C. After that, cells were lysed using liquid nitrogen and three repeated cycles of freeze-thawing. Then, samples were centrifuged at 12,000 × *g* for 20 min at 4 °C. Supernatants were denatured at 100 °C for 10 min and kept at −20 °C until Western Blot analysis.

### Molecular docking

The crystal structure (PDB code: 4N1U^10^, resolution 1.6 Å) of human MTH1 in complex with TH588 was obtained from the RCSB protein data bank. The preparation of the protein structure was performed under Amber 10: EHT force field using the Quickprep module with the default parameters, which included adding hydrogen atoms, repairing the missing residues and setting up the protonation states of the ionizable residues with pKa = 7. The ligand structure was prepared under Amber 10: EHT force field by energy minimization and conformational search, which supplied us the conformational database of ligands for the further docking studies. Then, all of the conformations were docked into the binding pocket of human MTH1 with the default parameters, using the method of flexible docking. The docking structures were scored by GBVI/WSA dG and held 20 docking poses in the case of parameters by default.

### Molecular dynamic simulation

The four residues Asp119, Asp120, Asn33 and Trp117 were manually mutated to Ala by the protein builder module in MOE 2015.10 package. Then, the mutations systems of D119A, D120A, N33A, W117A were balanced by 30 ns molecular dynamic (MD) simulations, using AmberTools14^62^ and NAMD^63^ packages. Both the small molecules and receptor of the complex were prepared with AmberTools14, and the force field of small molecules and receptor were GAFF and FF14SB, respectively. Besides, AM1-BCC charge was used to obtain the atomic charges of small molecule by the antechamber module. The complex was solvated with a box of water with the distance of 12 Å and sodium ions were added to neutralize the system by the leap module. At last, topology and coordinate parameter files were generated by AmberTools14. Then the MD simulation was performed by the NAMD package. Firstly, the energy minimization for 2000 steps was performed to minimize the solvent and the side chain of protein. The minimization for 3000 steps without any constraints was applied to minimize the whole system. Then, the temperature of the system was gradually heated from 0 to 300 K over 60 ps. Subsequently, the system equilibrated in NVT ensemble over 40 ps and in NPT ensemble over 50 ps, respectively. In the end, 30 ns MD simulations were carried out for each system in NPT ensemble at 1 atm.

### MM/PBSA binding free energy calculations

After the MD simulations of mutation systems, the obtained stable MD trajectory for each wild-type and mutation systems were used to assess the binding free energy (Δ*G*_bind_) using the MM/PBSA module in AmberTools14^62^ package. Δ*G*_bind_ can be calculated as:

$${{\Delta G}}_{{\mathrm{bind}}} = {{G}}_{{\mathrm{complex}}}-\left( {{{G}}_{{\mathrm{receptor}} + }{{G}}_{{\mathrm{ligand}}}} \right)$$

$${{\Delta G}}_{{\mathrm{bind}}} \approx {{\Delta E}}_{{\mathrm{MM}}} + {{\Delta G}}_{{\mathrm{sol}}}-{{T\Delta S}}$$

$${{\Delta G}}_{{\mathrm{sol}}} = {{\Delta G}}_{{\mathrm{polar(PB/GB)}}} + {{\Delta G}}_{{\mathrm{nonpolar(SA)}}},$$

where Δ*E*_MM_, Δ*G*_sol_, and −*T*Δ*S* are the molecular mechanics free energy, the solvation free energy, and the entropy term, respectively. Δ*G*_sol_ is composed of the polar contribution (Δ*G*_PB/GB_) and the nonpolar contribution (Δ*G*_SA_). As for MM/PBSA calculation, our work is to calculate relative binding free energy (ΔΔ*G*_bind_) for MI-743-MTH1 complex by comparing native and mutated systems, ΔΔ*G*_bind_ can be calculated as:

$${{\Delta \Delta G}}_{{\mathrm{bind}}} = {{\Delta G}}_{{\mathrm{bind}}}\left( {{\mathrm{mutant}}} \right)-{{\Delta G}}_{{\mathrm{bind}}}\left( {{\mathrm{wild - type}}} \right).$$

Entropy terms corresponding to native and mutated complexes with the same ligand may be similar and cancel out in the process of estimating the effect of four mutations systems on relative binding free energy. In addition, four mutations did not cause significant changes in binding modes conformations of ligands. Therefore, such entropy calculation is considered as neglected in the process of estimating ΔΔ*G*_bind_ values^64^. Here, 200 snapshots extracted from the last 5 ns stable MD trajectory were used to calculate Δ*E*_MM_, Δ*G*_PB_ and Δ*G*_SA_.

### Cell Viability Assay

The cell viability was determined by 3-(4,5-dimethylthiazol-2-yl)-2,5-diphenyltetrazolium bromide (MTT) assay, according to the manufacturer’s instructions. 1 day before treatment, cells were seeded into 96-well plates at a concentration of 2–5 × 10^3^ cells per well, followed by treatment with fresh medium containing serial dilutions of candidate compounds for 48 or 72 h, 20 μL of MTT solution (5 mg/mL in PBS) was then added to each well. After treatment, the plates were incubated at 37 °C for another 4 h. Subsequently, the MTT solution was removed and 150 μL of DMSO was added to each well for 10 min, and then the plates were shaken to dissolve the dark blue crystal (formazan), the absorbance was detected at 570 nm using a BioTek microplate reader. The data were analyzed using SPSS 20 software.

### Colony formation assay

The day before treatment, cells were seeded into six-well plates at a concentration of 1 × 10^3^ cells per well, and then the media were replaced with fresh media containing serial concentrations of candidate compounds. After 7 days of treatment, the cells were fixed with 75% ethanol, stained with 0.1% crystal violet solution and captured by the microscopy (Leica). After that, the crystal violet crystals were dissolved by adding methanol. The absorbance at 595 nm was measured by a BioTek micro plate reader. In the end, the data were analyzed using the GraphPad Prism 5 software.

### Analysis of cellular apoptosis

For the cell morphology analysis and Hoechst 33258 staining, MGC-803 and HGC-27 cells were plated in 6-well plates (3~5 × 10^5^ cells/mL) and incubated at 37 °C for 16–20 h. Complete medium (blank) and compound MI-743 at 2, 5 and 10 µM were added and then incubated for 48 h. Next, the cells were fixed for 20 min in cold methanol, stained for 30 min at 37 °C with 1 µg/mL Hoechst 33258 and examined under Nikon Eclipse TE 2000-S fluorescence microscope.

For the analysis of apoptosis by flow cytometry, MGC-803, HGC-27 and GES-1 cells were seeded into 6-well plates (1–1.5 × 10^6^ cells/mL) and incubated at 37 °C overnight. Then, fresh media, containing compound MI-743 at 0, 2, 5 and 10 µM, were added for 48 h. Cells were then harvested and washed twice with PBS. The Annexin-V-FITC/PI apoptosis kit was used to detect apoptotic cells, according to the manufacturer’s instructions. The cells were collected for each sample by the FACS Calibur flow cytometer (BD Biosciences) and the data were analyzed by Flow Jo-V10.

### Western Blot analysis

MGC-803, HGC-27 and GES-1 cells were seeded into 100mm^2^ plastic dishes(1 × 10^5^ cells/well) and incubated overnight prior to the addition of compound MI-743 at 1, 2, 4, 8 and 12 µM for 48 h or compound MI-743 at 12 µM for 12, 24, 36 and 48 h, respectively. After treatment, the cells were harvested and lysed with RIPA cell lysis buffer, containing protease inhibitor cocktail. Then, the cells were centrifuged at 12,000 × *g* for 20 min at 4 °C. The supernatant were collected and the concentration was determined by a Bicinchoninic Acid (BCA) Protein Assay kit, denatured at 100 °C for 10 min, separated by SDS−PAGE and transferred to 0.22 μm nitrocellulose membranes. After blocking by PBS containing 5% skim milk for 2 h at room temperature, the membranes were probed with the appropriate primary antibodies (1:500~1:1000) at 4 °C overnight, followed by the treatment of horseradish-peroxidase-conjugated secondary antibody (1:10000) at room temperature for 2 h. Subsequently, the membranes were washed with PBST, examined by enhanced chemiluminescence and the data was determined using Image J software.

### siRNA experiments

Cells were seeded in 6-well or 96-well plates at approximately 30% confluency for 24 h before siRNA treatment. Then, medium was taken away and transfections were performed with Lipofectamine® RNAiMAX Reagent, according to manufacturer’s instructions. After 3 days, cells were collected and used for other relative experiments. Two different siRNA duplexes targeting two specific sequences of MTH1 and a scrambled siRNA as a negative control were synthesized by GenePharma(Shanghai, China) as described previously. The following siRNA sequences were used: MTH1 siRNA #1 5′-GACGACAGCUACUGGUUUC-3′; MTH1 siRNA #2 5′-CGACGACAGCUACUGGUUU-3′^10^.

### Comet assay

MGC-803 and HGC-27 cells were seeded into 6-well plates(1.5 × 10^5^ cells/well) and cultured overnight. For siRNA experiments, the control siRNA and MTH1 siRNA were transfected into cells. For compounds experiments, cells were treated with candidate compounds MI-743 for 48 h before collection. For the H_2_O_2_ control, cells were treated with 100 µM H_2_O_2_ for 10 min. Subsequently, cells were harvested, washed and resuspended with PBS. After counting, cells were mixed with 1.2% low melting-temperature agarose at 37 °C. The mixtures were layered onto pre-warmed glass slides, which were pre-coated with 1% agarose. After adding coverslips, slides were kept in 4 °C immediately for 10-15 min, followed by immersing in pre-chilled lysis buffer(2.5 M NaCl, 0.1 M EDTA, 10 mM Tris-HCL pH 7.7, 1% Triton X-100, 1% DMSO)for 5-8 h. Then, slides were washed three times with enzyme reaction buffer (40 mM HEPES pH 8.0, 0.1 M KCl, 0.5 mM EDTA and 0.2 mg/mL BSA) and incubated for another 45 min in pre-chilled alkaline electrophoresis buffer (50 mM NaOH, 1 mM EDTA, 1% DMSO, pH12.8). After electrophoresis for 30 min at 25 V, slides were neutralized with 0.4 M Tris-HCl pH 7.0 for 1 h and stained with 1‰ EB for 5 min. At last, images were acquired using fluorescence microscope and analysised by CometScore software.

### 8-oxo-dG assay

Intracellular 8-oxo-dG levels were measured by staining with Cy3-conjugated avidin^65^. Cells were seeded on coverslips in 24-well plates (1–1.5 × 104 cells/well) overnight, and then transfected with candidate compounds. After 48 h, the cells were fixed with ice-cold methanol in Tris-buffered saline (TBS) for 15 min and blocked, using TBS with 0.1% Triton X-100 and 15% fetal bovine serum for 1 h. Then, the cells were stained with Cy3-conjugated avidin (0.5 µg/mL) for 1 h at room temperature. DNA was counterstained with 4,6-diamidino-2-phenylindole(DAPI). Images were acquired in a Nikon Eclipse TE 2000-S fluorescence microscope.

### Animals and tumor xenograft model

All animal experiments were conducted according to the guidelines of the Institutional Animal Care and Use Committee of Zhengzhou University. Four or five-week-old female BALB/c nude mice, weighing 18–21 g were purchased from Hunan Slack Scene of Laboratory Animal Company, LTD (Hunan, China). They received abundant food and water and were housed in a laminar flow under sterilized condition. 200 µL MGC-803 cells at the concentration of 5 × 10^7^/mL were injected into the right scapular region of the mice. When most of the tumor volumes reached 100 mm^3^, mice were randomly divided into oil (solvent) group and MI-743(40 mg/kg) treatment group. Then, they received gavage daily for 25 days. The body weight was weighed every 2 days and the tumor volumes were measured every 4 days. At the 25th day, the tumors were surgically removed and weighed. The tumor size was determined by vernier caliper measurements and the tumor and major organs were fixed in 4% buffered paraformaldehyde and paraffin-embedded for hematoxylin and eosin (HE) staining, immunohistochemistry (IHC) and TUNEL assay.

### Statistical Analysis

Data were expressed as mean ± SD. Statistical differences in two groups were performed by studentʹs *t* test using GraphPad Prism 5 software. One-way ANOVA analysis was used for multiple group comparison by SPSS software. *P* values of 0.05 or less were considered statistically significant.

## Supplementary information

Supplemental file

Supplemental figure1

Supplemental figure2

Supplemental figure3

Supplemental figure4

Supplemental figure5

Supplemental figure6
