# Correction: Potent and specific MTH1 inhibitors targeting gastric cancer

**DOI:** 10.1038/s41419-020-03010-x

**Published:** 2020-10-16

**Authors:** Wenjuan Zhou, Liying Ma, Jing Yang, Hui Qiao, Lingyu Li, Qian Guo, Jinlian Ma, Lijuan Zhao, Junwei Wang, Guozhong Jiang, Xiangbin Wan, Mariusz Adam Goscinski, Lina Ding, Yichao Zheng, Wencai Li, Hongmin Liu, Zhenhe Suo, Wen Zhao

**Affiliations:** 1 State Key Laboratory of Esophageal Cancer Prevention and Treatment; Key Laboratory of Advanced Pharmaceutical Technology Ministry of Education of China; School of Pharmaceutical Sciences, Zhengzhou University, 100 Kexue Avenue, Zhengzhou, Henan 450001 China; 2 Department of Pathology, Oslo University Hospital, Faculty of Medicine, University of Oslo, Oslo, 0379 Norway; 3 Department of Pathology, The First Affiliated Hospital, Zhengzhou University, Zhengzhou, Henan 450001 China; 4 Department of General Surgery, Henan Provincial People’s Hospital, Zhengzhou, Henan 450001 China; 5 Department of Urology, The Norwegian Radium Hospital, Oslo University Hospital Oslo, 0379 Norway

**Keywords:** Targeted therapies, Gastric cancer

Correction to: *Cell Death & Disease*

10.1038/s41419-019-1665-3, published online 4 June 2019

Since online publication of this article, the authors noticed that there were errors in Figs. 4 and 5. In Fig. 4, the colouring was omitted from the key for Fig. 4d, g, h, l and m, and an incorrect image was used for OGG1 during the compilation of Fig. 4d. In Fig. 5, incorrect images were used for GAPDH and Procaspase 3 in Fig. 5i, 5j, respectively. The corrected Figures are provided below. The authors confirm that these errors do not affect the conclusions of the article. The authors apologise for any inconvenience this may have caused.

**Figure:**
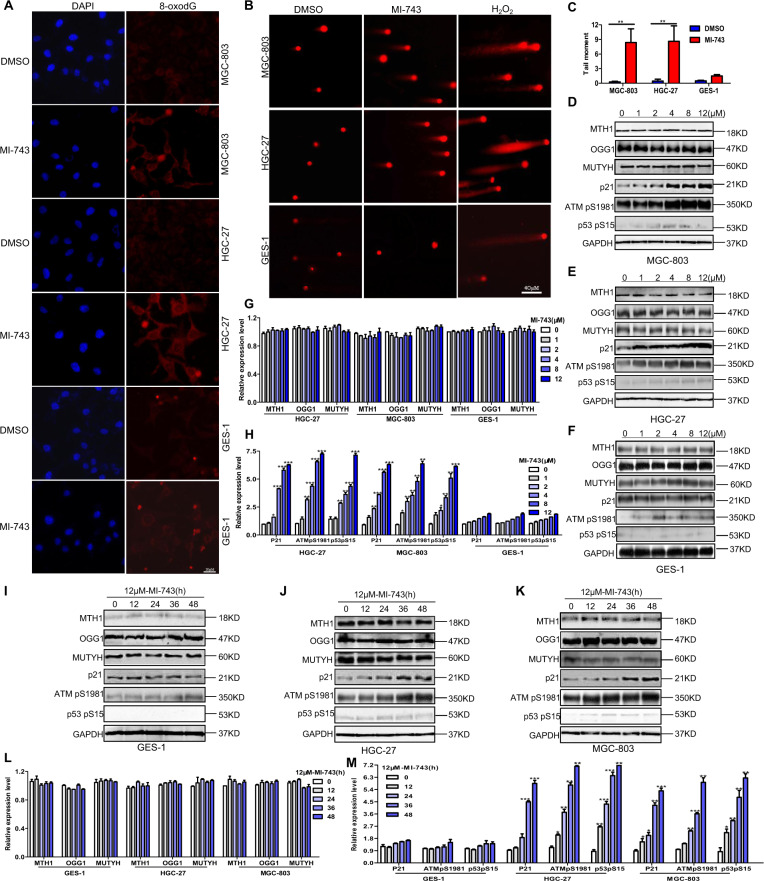
Fig. 4

**Figure:**
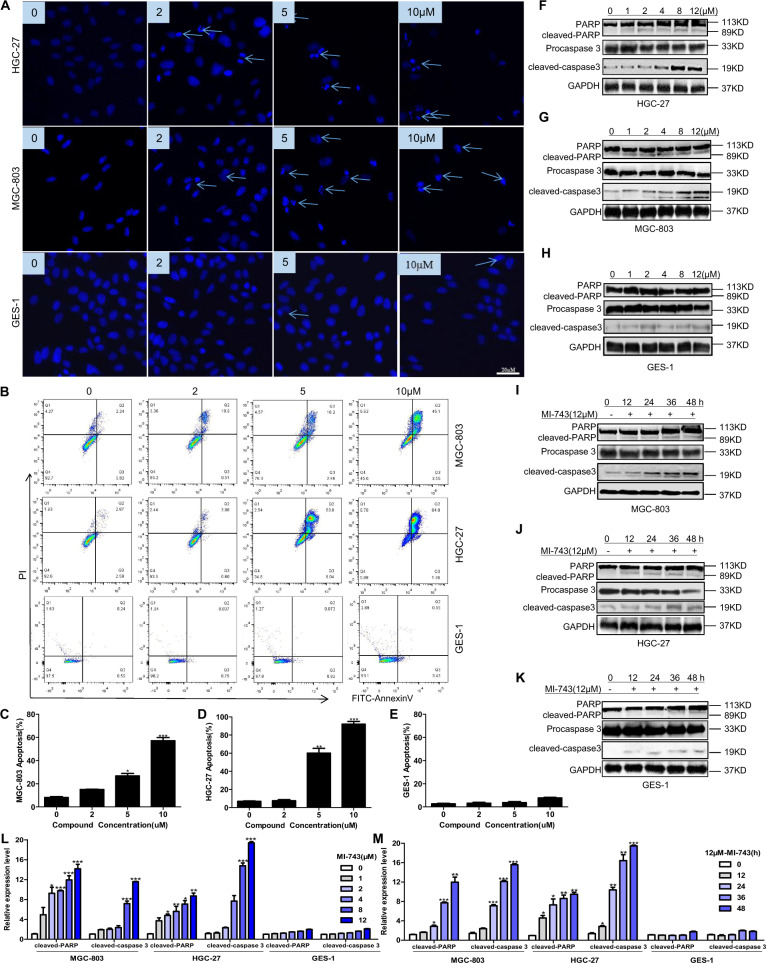
Fig. 5

