# Supplementary material for: Potent and specific MTH1 inhibitors targeting gastric cancer
Source: Cell Death Dis. 2019 Jun 4;10(6):434. doi: 10.1038/s41419-019-1665-3 (PMC6547740; doi:10.1038/s41419-019-1665-3)
Supplement: Supplementary file 1 — Supplemental file [file 41419_2019_1665_MOESM1_ESM.docx]

**SUPPLEMENTARY METARIALS**

**Chemistry**

The general route for the synthesis of the target pyrimidine-thiourea hybrids is depicted in **Scheme 1**. 6-aryl-5-cyano-2-thiouracils **4a**-**b** were prepared via prolonged heating of aldehydes **1a-b**, ethylcyanoacetate **2** and thiourea **3** in ethanol, in the presence of potassium carbonate[^1^](#_ENREF_1). Compounds **4a**-**b** were allowed to react with propargyl bromide in the presence of phosphorous oxychloride, in dioxane to yield the target derivatives **5a**-**b**. Compounds **9b** were prepared via click reaction of compound **5b** with **8**. **5a** and **10b** were then reacted with the corresponding amines to obtain compounds **MI-743** and **MI-401**.

**Scheme 1.** Reagents and conditions: **a**: absolute ethanol, absolute K_2_CO_3,_ reflux, 10h; **b**: i) propargyl bromide, dioxane, reflux; ii) phosphorous oxychloride, reflux, 1h; **c**: appropriate amine, absolute ethanol, reflux, 6h. **d**: i) Oxalyl chloride, 1,2-dichloroe, 90°C; ii) 2-aminopyridine, 0°C; **e**: NaN_3_, Acetone-H_2_O (4:1), reflux; **f**: CuSO_4_·5H_2_O, Sodium ascorbate, THF-H_2_O (1:1), rt.

**General Experimental**

The reaction process was monitored by TLC with silica gel plates (thickness 250μm, Indicator F-254). The target analogues were purified by column chromatography with silica gel (300 meshes). Melting points were determined on an electro thermal melting point apparatus and were reported uncorrected. The structures of intermediates and target analogues were characterized by NMR (400 and 100 MHz) in DMSO-d_6_ with TMS as an internal standard and HRMS. The purity of all biologically evaluated compounds was determined to be >95% by reverse phase high performance liquid chromatography (HPLC) analysis.

**Experimental Procedures and Analytical Data**

General procedure for the synthesis of compounds **5a-b**

A mixture of the appropriate 2-mercapto-dihydroyrimidine derivatives **4a-b** (1mmol), the propargyl bromide (1mmol) and anhydrous potassium carbonate (1mmol) were refluxed in dry dioxane. Upon completion, as judged by TLC, phosphorous oxychloride was added dropwise with stirring, while maintaining the temperature of the reaction mixture. Stirring was continued for additional 1h. The cooled reaction mixture was poured on crushed ice and the separated solid was filtered off, washed with water, dried and crystallized from aqueous ethanol to yield the pure product.

**4-chloro-6-(2-oxo-2H-chromen-6-yl)-2-(prop-2-yn-1-ylthio)pyrimidine-5-carbonitrile(5a)**

Yellow solid. Mp: 188–189°C. 1H NMR (400 MHz, DMSO-d6, ppm) δ 8.42 (d, J = 2.2 Hz, 1H, -CH=), 8.26 (dd, J = 8.7, 2.2 Hz, 1H, -CH=), 8.21 (d, J = 9.6 Hz, 1H, Ar-H), 7.66 (d, J = 8.7 Hz, 1H, Ar-H), 6.63 (d, J = 9.6 Hz, 1H, Ar-H), 4.15 (d, J = 2.6 Hz, 2H, -CH2-), 3.27 (t, J = 2.6 Hz, 1H, ≡C-H). 13C NMR (100 MHz, DMSO-d6, ppm) δ 159.43, 155.46, 143.85, 132.05, 131.23, 129.45, 118.77, 117.25, 116.81, 115.59, 93.23, 79.17, 74.28, 19.28. HR-MS (ESI), calcd. C17H8ClN3O2S, [M + Na]+ m/z: 375.9923, found: 375.9925.

**4-(4-bromophenyl)-6-chloro-2-(prop-2-yn-1-ylthio)pyrimidine-5-carbonitrile (5b)**

Yield 80.5%. White solid. Mp: 137–138°C.^1^H NMR (400 MHz, CDCl_3_, δ, ppm)δ 8.08 – 7.96 (m, 2H, Ar-H), 7.84 – 7.60 (m, 2H, Ar-H), 4.00 (d, *J* = 2.6 Hz, 2H, -CH_2_-), 2.27 (t, *J* = 2.6 Hz, 1H, ≡C-H).^13^C NMR (100 MHz, CDCl_3_, δ, ppm) δ 174.24, 167.54, 164.06, 132.85, 132.39, 130.78, 127.98, 114.24, 101.19, 78.09, 71.65, 20.40. HR-MS (ESI): Calcd. C_14_H_8_BrClN_3_S, [M+H]^+^m/z: 363.9311, found: 363.9314.

**Procedure to synthesize compounds 8**

To a solution of compound **7** (2.0g, 21.5mmol ) in anhydrous 1,2-dichloroethane (20mL) was added oxalyl chloride (2mL) at 0 °C, then refluxed on oil bath at 90 °C for 4 h. The reaction mixture was then cooled to room temperature. 2-aminopyridine (21.5mmol) was added into the reaction mixture. The reaction mixture was stirred for another 5 min. Upon completion, the solid was filtrated and washed with 1,2-dichloroethane to yield the pure product. Yield 86.5%. Yellow solid. Mp: 159–160°C.^1^H NMR (400 MHz, DMSO-d_6_, δ, ppm)δ 11.27 (s, 1H, NH, D_2_O exchangeable), 10.71 (s, 1H, NH, D_2_O exchangeable), 8.39 – 8.26 (m, 1H, ArH), 7.92 (ddd, *J* = 12.4, 10.2, 5.0 Hz, 2H, ArH), 7.29 – 7.09 (m, 1H, ArH), 4.47 (s, 2H, CH_2_). ^13^C NMR (100 MHz, DMSO-d6, δ, ppm): δ 11.27, 10.71, 8.35, 8.35, 8.34, 8.34, 8.34, 7.97, 7.95, 7.93, 7.92, 7.91, 7.90, 7.89, 7.88, 7.22, 7.22, 7.21, 7.20, 7.20, 7.19, 7.19, 4.47. HR-MS (ESI): Calcd. C_8_H_9_ClN_3_O_2_, [M+H]+m/z: 214.0383, found: 214.0379.

**Procedure to synthesize compounds 9**

To a magnetically stirred solution of compound **8** (1.2g, 5.6mmol) in CH_3_CN (10mL), sodium azide (0.73 g, 11.2mmol) was added carefully and the reaction mixture was refluxed for 8h. Upon completion, the reaction mixture was concentrated under vacuum. The residue was dissolved in EtOAc (30mL) and washed with water, brine, dried over anhydrous Na2SO4 and concentrated under vacuum to give compound **9** (0.60 g, yield 92.0%), which was used in the next reaction without further purification. Yield 90.5%. White solid. Mp: 159–160°C.^1^H NMR (400 MHz, DMSO-d_6_, δ, ppm)δ 11.01 (s, 1H, NH, D_2_O exchangeable), 10.52 (s, 1H, NH, D_2_O exchangeable), 8.42 – 8.22 (m, 1H, ArH), 7.93 (d, *J* = 8.1 Hz, 1H, ArH), 7.89 – 7.71 (m, 1H, ArH), 7.30 – 7.02 (m, 1H, ArH), 4.21 (s, 2H, CH_2_). ^13^C NMR (100 MHz, DMSO-d6, δ, ppm): δ 11.27, 10.71, 8.35, 8.35, 8.34, 8.34, 8.34, 7.97, 7.95, 7.93, 7.92, 7.91, 7.90, 7.89, 7.88, 7.22, 7.22, 7.21, 7.20, 7.20, 7.19, 7.19, 4.47. HR-MS (ESI): Calcd. C_8_H_9_N_6_O_2_, [M+H]+m/z: 221.0787, found: 221.0789.

**General procedure for the synthesis of compounds 10**

In a round-bottom flask equipped with a magnetic stirred bar, **5a** (5mmol), **9** (5mmol), CuSO_4_^.^5H_2_O (62mg, 0.25mmol), sodium ascorbate (100 mg, 0.5mmol), THF (20 mL) and dH_2_O (20 mL) were added. The resulting mixture was stirred at room temperature. Upon completion, dH_2_O was added and the reaction mixture was extracted with EtOAc. The combined organic layer was washed with brine, dried over anhydrous Na_2_SO_4_ and concentrated under vacuum to afford the crude product. The crude product was recrystallized from acetone to yield the pure product.

**2-(4-((4-(4-bromophenyl)-6-chloro-5-cyanopyrimidin-2-yl)thiomethyl)-1H-1,2,3-triazol-1-yl)-N-(pyridin-2-ylcarbamoyl)acetamide (10)**

Yield 68.5%. Yellow solid. Mp: 167–168°C. ^1^H NMR (400 MHz, DMSO-d_6_, δ, ppm)δ 11.41 (s, 1H, NH, D_2_O exchangeable), 10.60 (s, 1H, NH, D_2_O exchangeable), 8.40 (s, 1H, ArH), 8.12 (s, 1H, ArH), 8.00 (d, *J* = 7.9 Hz, 3H, ArH), 7.91 (s, 1H, ArH), 7.68 (dd, *J* = 24.7, 7.7 Hz, 2H, ArH), 7.22 (s, 1H), 5.50 (s, 2H, Ar-CH_2_), 4.63 (s, 2H, S-CH_2_).^13^C NMR (100 MHz, DMSO-d_6_, δ, ppm) δ 166.58, 166.07, 163.11, 161.44, 150.59, 140.22, 137.89, 137.18, 134.40, 133.58, 133.47, 131.57, 131.18, 129.52, 129.24, 126.14, 116.15, 93.87, 52.91, 25.67. HRMS (ESI) calcd for C_22_H_16_BrClN_9_O_2_S [M+H]^+^: m/z: 584.0020, found: 584.0023.

**General procedure for the synthesis of compounds MI-743 and MI-401**

To a well stirred solution of the appropriate amine (5.0mmol) in absolute ethanol (10mL), equimolar amount of a solution of compounds **5a** or **10** (5.0mmol) in absolute ethanol (10mL) was added. The reaction mixture was stirred for 1.5h at room temperature, then heated under reflux for additional 5h. Upon completion, the precipitated product was filtered off and washed with ethanol to afford the crude product. The crude product was recrystallized from ethanol to yield the appropriate pure product.

**2-(5-cyano-6-(2-oxo-2H-chromen-6-yl)-2-(prop-2-yn-1-ylthio)pyrimidin-4-yl)hydrazine-1-carbothioamide (MI-743)**

Yield 56.3%. Yellow solid. ^1^H NMR (400 MHz, DMSO-d_6_, δ, ppm)δ 12.75 (s, 1H, NH, D_2_O exchangeable), 8.27 (s, 1H), 8.22 (d, J = 9.5 Hz, 1H), 8.12 (d, J = 8.6 Hz, 1H), 7.60 (d, J = 8.5 Hz, 1H), 6.60 (d, J = 9.5 Hz, 1H), 5.21 (s, 2H, NH, D_2_O exchangeable), 4.05 (s, 2H, S-CH_2_), 3.16 (s, 1H, ≡C-H). ^13^C NMR (100 MHz, DMSO-d_6_, δ, ppm) δ 165.85, 159.70, 154.89, 144.38, 132.66, 132.51, 129.65, 118.91, 116.80, 116.65, 80.47, 73.17, 18.72. HRMS (ESI) calcd for C_18_H_11_N_6_O_2_S_2_ [M-H]^-^: m/z: 407.0385, found: 407.0383.

**2-(4-((4-(4-bromophenyl)-5-cyano-6-(p-tolylamino)pyrimidin-2-yl)thiomethyl)-1H-1,2,3-triazol-1-yl)-N-(pyridin-2-ylcarbamoyl)acetamide (MI-401)**

Yield 68.5%. Yellow solid. Mp: 155–156°C. 1H NMR (400 MHz, DMSO-d6, δ, ppm)δ 11.30 (s, 1H, NH, D2O exchangeable), 10.43 (s, 1H, NH, D2O exchangeable), 9.94 (s, 1H, NH, D2O exchangeable), 8.32 (s, 1H), 8.00 – 7.87 (m, 3H, ArH), 7.84 (d, J = 7.1 Hz, 1H, ArH), 7.65 (d, J = 7.9 Hz, 2H, ArH), 7.57 (s, 1H, ArH), 7.43 (d, J = 7.6 Hz, 2H, ArH), 7.17 (d, J = 8.1 Hz, 3H, ArH), 5.41 (s, 2H, Ar-CH2), 4.37 (s, 2H, S-CH2), 2.29 (s, 3H, Ar-CH3). 13C NMR (100 MHz, DMSO-d6, δ, ppm) δ 173.22, 169.25, 167.50, 160.69, 151.25, 150.54, 148.65, 139.13, 136.58, 135.27, 135.11, 135.06, 131.56, 131.08, 129.36, 129.15, 125.47, 124.88, 120.23, 116.30, 113.50, 85.08, 52.65, 25.56, 21.02. HRMS (ESI) calcd for C29H23BrN10NaO2S [M+Na]+: m/z: 677.0807, found: 677.0807.

**Supplementary figures:**

Supplementary Figure 1. The mRNA levels of MTH2, OGG1 and MUTYH in cancer cells. **(A)** The cancer cell lines: SMMC-7721, ZIP177, HepG2, EC109, EC9706, KYSE450, MGC-803, HGC-27 and MKN45, as well as normal cell lines: Het-1A, L02 and GES-1 were cultured and lysed. The mRNA levels of MTH2, MUTYH and OGG1 in these cell lines were determined by RT-PCR. GAPDH was used as control. At least three independent experiments were performed for each group. **(B)** The primer sequences of human MTH1, MTH2, MUTYH, OGG1 and GAPDH for RT-PCR. Data are presented as means ± SD. Three individual experiments were performed for each group. The symbols *, ** or *** stand for P<0.05, P<0.01 or P<0.001 compared with the controls.

Supplementary Figure 2. The expression levels of NUDT1 (MTH1) in stomach adenocarcinoma(STAD) and non-tumor tissues from TCGA database were analyzed by websites: UALCAN (http://ualcan.path.uab.edu/index.html)**(A)** and Oncomine (https://www.oncomine.org)**(B)**. **(C)** The correlation of NUDT1(MTH1) expression level with patient survival rate and overall survival days. The expression levels of NUDT1(MTH1) in esophageal carcinoma (ESCA)**(D)**, liver hepatocellular carcinoma(LIHC)**(E)** and their corresponding non-tumor tissues from TCGA database，which were analyzed by UALCAN (<http://ualcan.path.uab.edu/index.html>)**.** The symbols *, ** or *** stand for P<0.05, P<0.01 or P<0.001 compared with the controls.

Supplementary Figure 3. The expression levels of NUDT1(MTH1) in TCGA gastric subtypes**(A)**. The correlation of NUDT1(MTH1) expression level in stomach adenocarcinoma(STAD) with patient gender**(C)** and age**(B).**

Supplementary Figure 4. Screening of MTH1 inhibitors in vitro. **(A)** The plasmids of pET28a-MTH1 and its mutant pET28a-E56A were induced and expressed by IPTG and purified by Ni-beads column. The purity and abundance of MTH1 and MTH1-E56A were determined by SDS-PAGE and Commassie Blue staining. **(B)** The activities of MTH1 and its mutant protein E56A were determined by the amount of the green associated complex after the products of inorganic pyrophosphate’s reaction with malachite green and ammonium molybdate, at the absorbance of 630nm, using PerkinElmer Envision micro-plate reader. **(C)** The MTH1 activity screening system in vitro was optimized, using TH588 as a positive control.

Supplementary Figure 5. Binding mode of compound MI-743 and MI-401 in MTH1 pocket. Predicted binding mode of compound MI-743**(A)** in MTH1 binding pocket (type B) and compound MI-401**(B)** in MTH1 binding pocket(PDB code: 4N1U). Compound MI-401 is shown as green stick. The residues associated with the compound are shown in white. Hydrogen bonds are shown in magenta dash lines. **(C)** The ligand-binding pocket surface of MTH1 and compound MI-401. Cyan area represents hydrophobic region and pink area represents hydrophilic region. The corresponding distances are given in Å. **(D)** MM/PBSA binding free energy estimated with the three systems obtained from the last 5 ns stable MD trajectory.

Supplementary Figure 6. siRNA#2 induced MTH1 knockdown in cancer cells. **(A)**The levels of MTH1 in these cell lines: SMMC-7721, SGC-7901, GES-1, HGC-27, MGC-803, MKN45, EC9706, EC109 and HepG2 were determined by Western Blot after siRNA#2 treatment. GAPDH was used as a loading control. **(B)** The viability of the above cells was determined by MTT assay, compared with those in the non-targeting siRNA(NT) treatment. Data are presented as means ± SD. Three individual experiments were performed for each group. The symbols *, ** or *** stand for P<0.05, P<0.01 or P<0.001 compared with the controls.

**Reference**

1. Ma, L. Y. *et al.* Design, Synthesis, and Structure-Activity Relationship of Novel LSD1 Inhibitors Based on Pyrimidine-Thiourea Hybrids As Potent, Orally Active Antitumor Agents. *Journal of medicinal chemistry*. **58**, 1705-1716 (2015).
