# Supplementary figures and images for: Potent and specific MTH1 inhibitors targeting gastric cancer
Source: Cell Death Dis. 2019 Jun 4;10(6):434. doi: 10.1038/s41419-019-1665-3 (PMC6547740; doi:10.1038/s41419-019-1665-3)

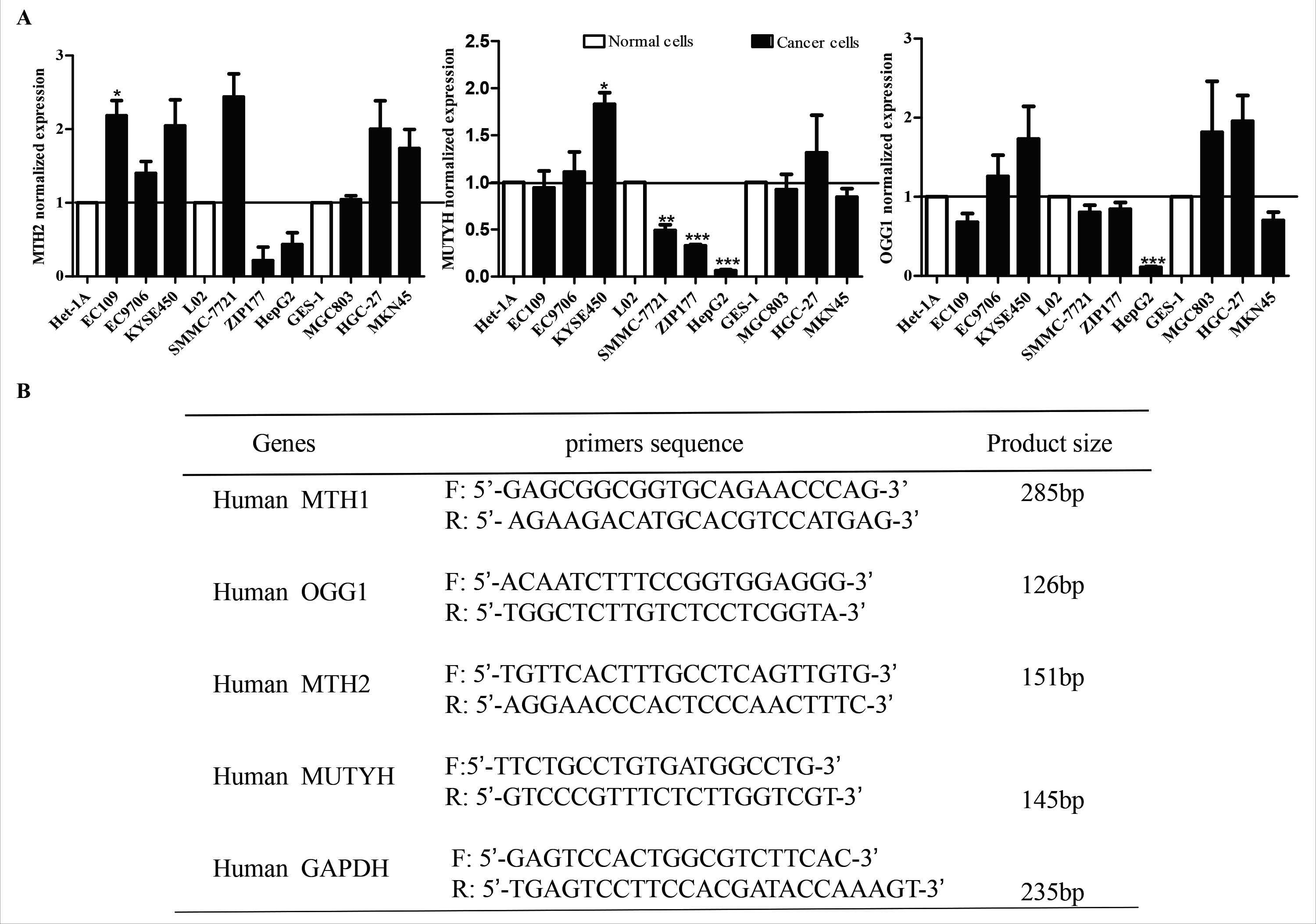

Supplement: Supplementary file 2 — Supplemental figure1 [file 41419_2019_1665_MOESM2_ESM.tif]

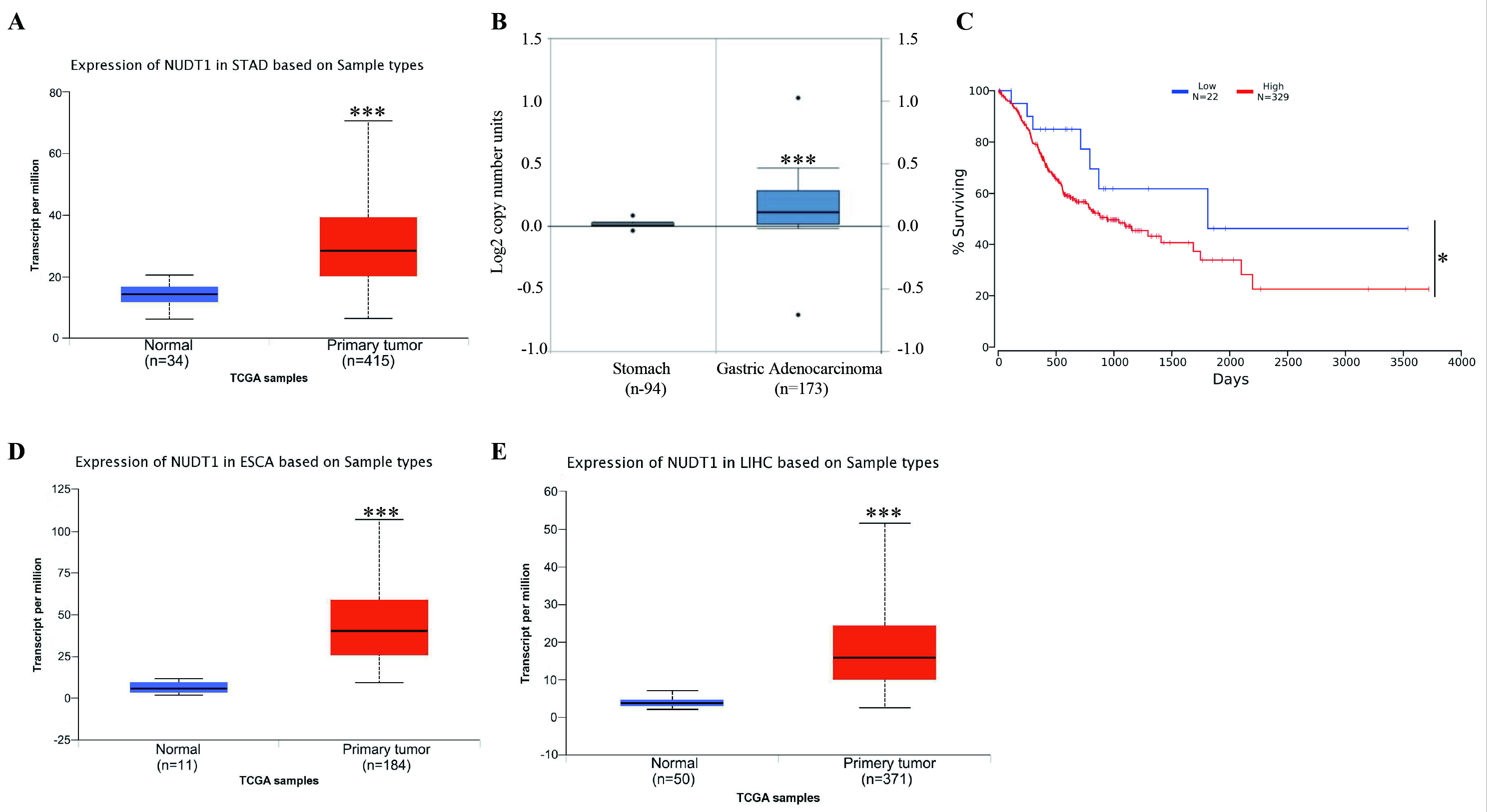

Supplement: Supplementary file 3 — Supplemental figure2 [file 41419_2019_1665_MOESM3_ESM.tif]

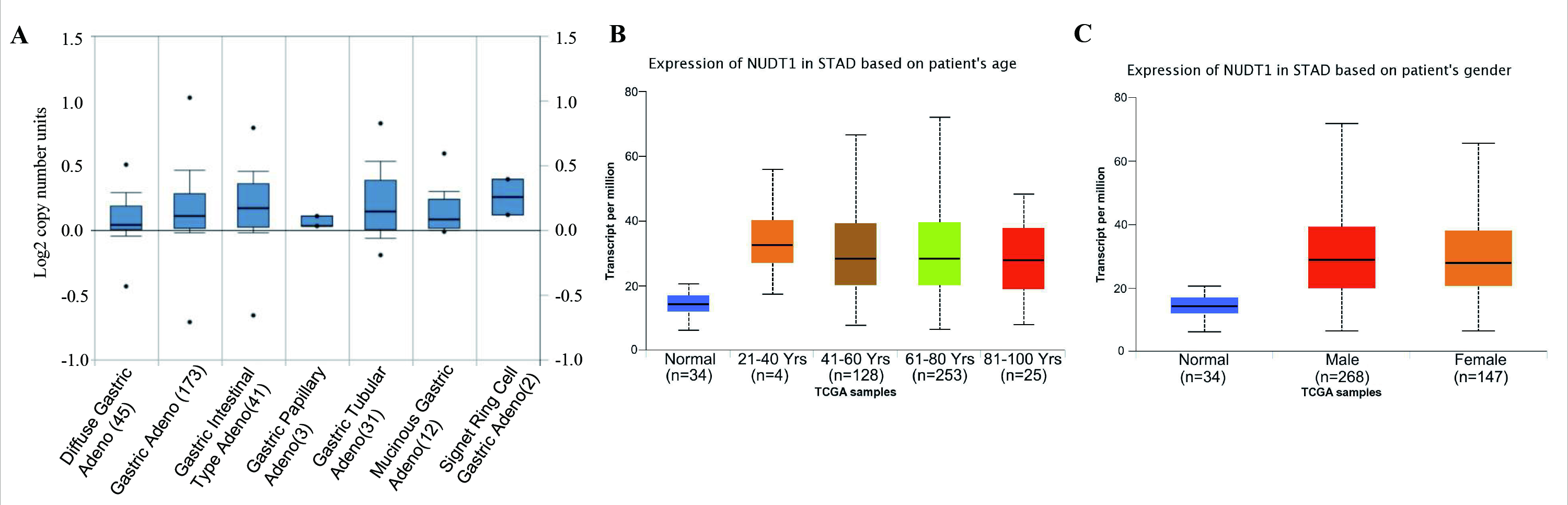

Supplement: Supplementary file 4 — Supplemental figure3 [file 41419_2019_1665_MOESM4_ESM.tif]

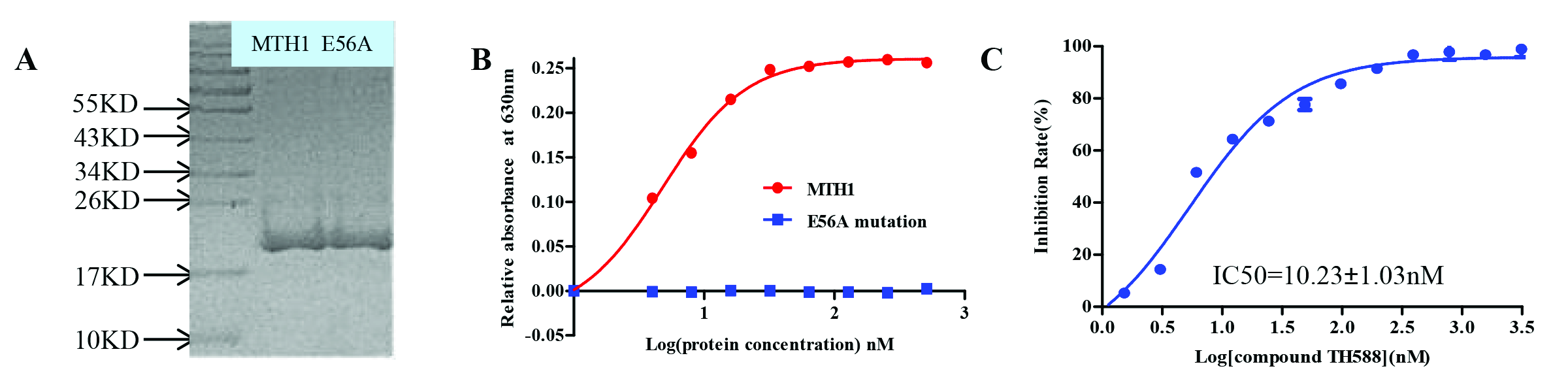

Supplement: Supplementary file 5 — Supplemental figure4 [file 41419_2019_1665_MOESM5_ESM.tif]

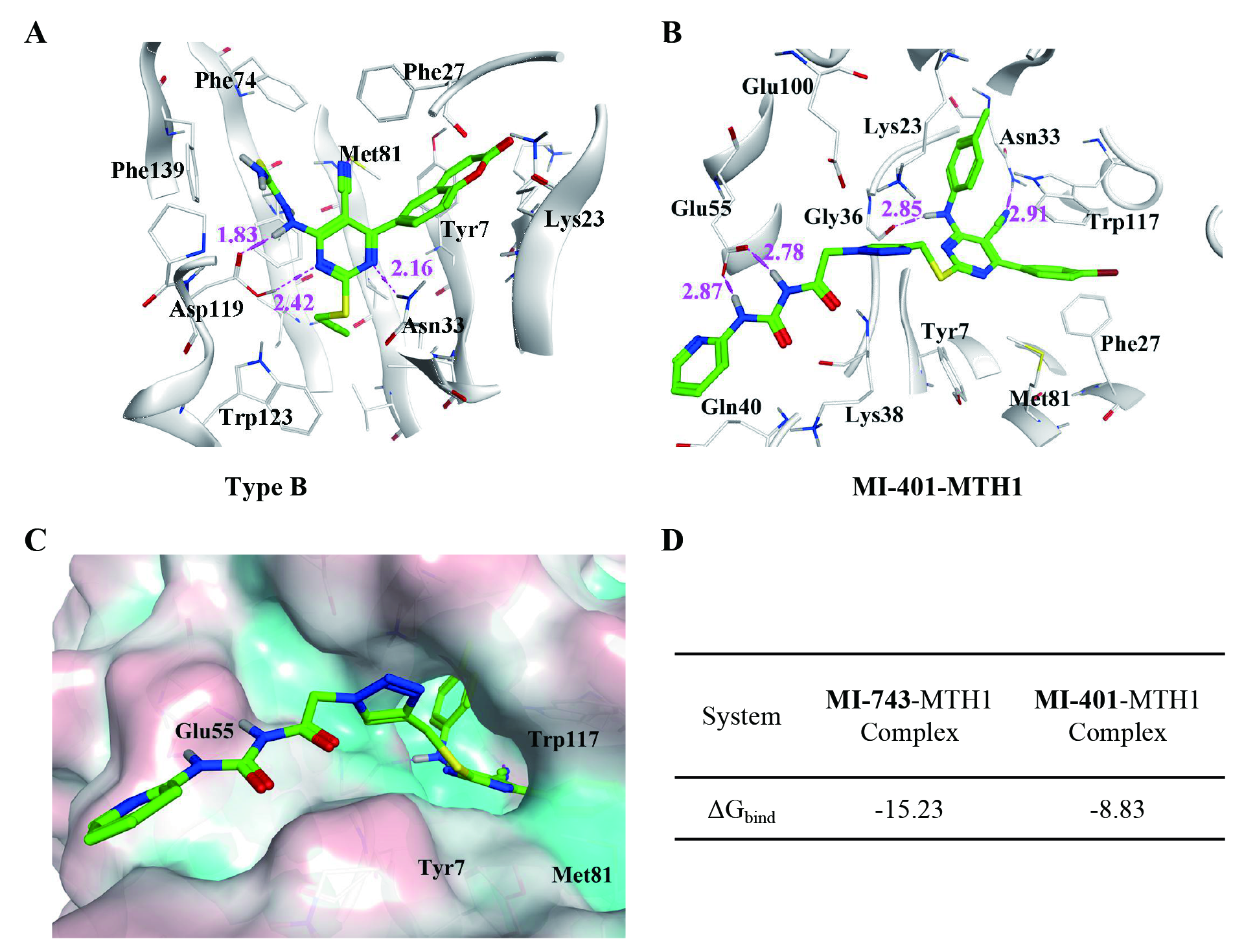

Supplement: Supplementary file 6 — Supplemental figure5 [file 41419_2019_1665_MOESM6_ESM.tif]

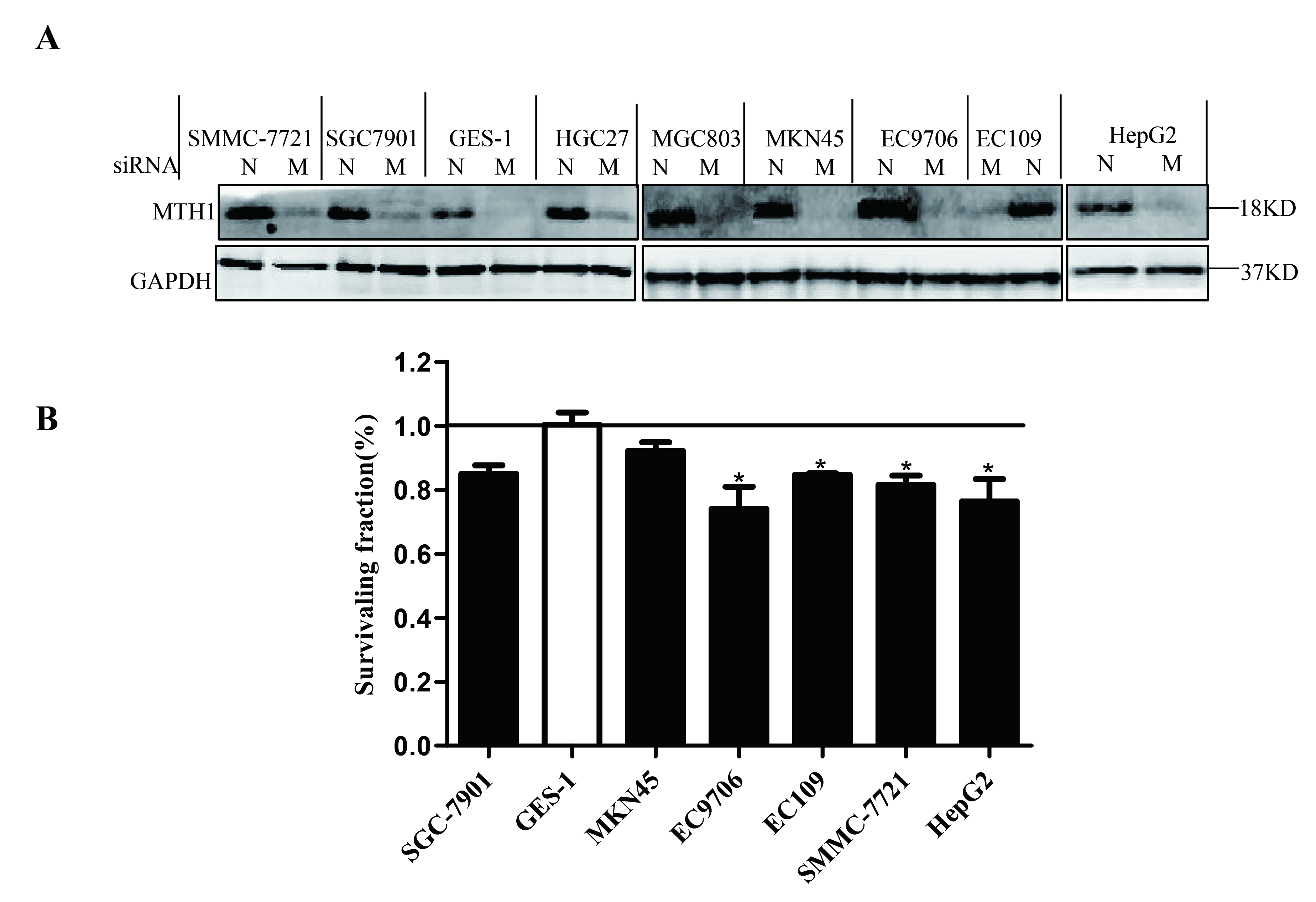

Supplement: Supplementary file 7 — Supplemental figure6 [file 41419_2019_1665_MOESM7_ESM.tif]
